# Optimizing Exciton and Charge-Carrier Behavior in Thick-Film Organic Photovoltaics: A Comprehensive Review

**DOI:** 10.1007/s40820-025-01852-8

**Published:** 2025-07-23

**Authors:** Lu Wei, Yaxin Yang, Lingling Zhan, Shouchun Yin, Hongzheng Chen

**Affiliations:** 1 Key Laboratory of Organosilicon Chemistry and Materials Technology of Ministry of Education, Zhejiang Key Laboratory of Organosilicon Material Technology, College of Materials, Chemistry and Chemical Engineering, Hangzhou Normal University, 311121 Hangzhou, People’s Republic of China; 2 State Key Laboratory of Silicon and Advanced Semiconductor Materials, Department of Polymer Science and Engineering, Zhejiang University, 310027 Hangzhou, People’s Republic of China

**Keywords:** Organic photovoltaics, Thick-film, Exciton, Charge-carrier, Photovoltaic performances

## Abstract

Organic photovoltaics (OPVs) have achieved remarkable progress, with laboratory-scale single-junction devices now demonstrating power conversion efficiencies (PCEs) exceeding 20%. However, these efficiencies are highly dependent on the thickness of the photoactive layer, which is typically around 100 nm. This sensitivity poses a challenge for industrial-scale fabrication. Achieving high PCEs in thick-film OPVs is therefore essential. This review systematically examines recent advancements in thick-film OPVs, focusing on the fundamental mechanisms that lead to efficiency loss and strategies to enhance performance. We provide a comprehensive analysis spanning the complete photovoltaic process chain: from initial exciton generation and diffusion dynamics, through dissociation mechanisms, to subsequent charge-carrier transport, balance optimization, and final collection efficiency. Particular emphasis is placed on cutting-edge solutions in molecular engineering and device architecture optimization. By synthesizing these interdisciplinary approaches and investigating the potential contributions in stability, cost, and machine learning aspects, this work establishes comprehensive guidelines for designing high-performance OPVs devices with minimal thickness dependence, ultimately aiming to bridge the gap between laboratory achievements and industrial manufacturing requirements.

## Highlights

- Research progress summary: Provides a systematic review of recent advancements in thick-film organic photovoltaics (OPVs) with a focus on molecular design and device engineering strategies.
- Efficiency enhancement strategies: Explores the mechanisms limiting efficiency in thick-film devices, analyzes exciton and charge-carrier dynamics, and identifies effective approaches to improve device performance.
- Industrialization contributions and outlook: Summarizes the potential contributions of thick-film OPVs to industrial applications and offers insights into future development directions (in stability, cost, and machine learning aspects).

## Graphical abstract

**Figure:**
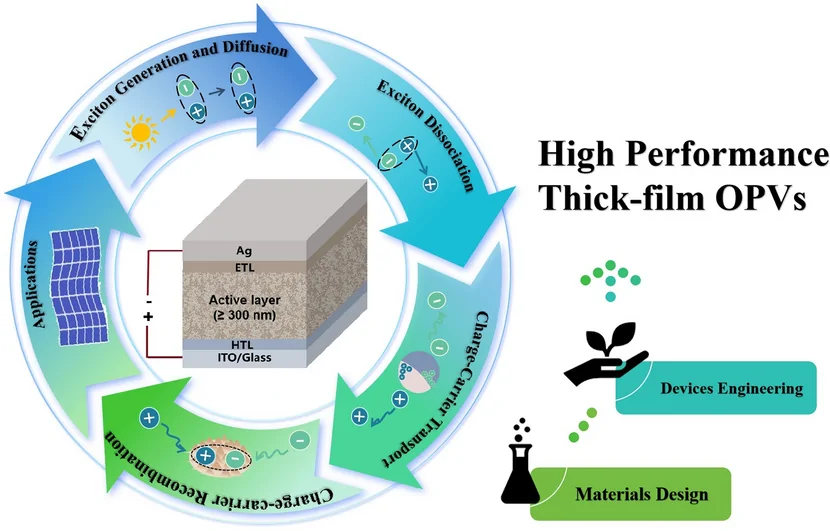

## Introduction

Organic photovoltaics (OPVs) have emerged as promising candidates for next-generation energy technologies due to their lightweight, flexible [1–15], semitransparent [16–30], and solution-processable characteristics [31–38]. However, their industrialization is currently limited by lower power conversion efficiencies (PCEs) compared to inorganic counterparts and challenges in scaling up [39–43]. The thickness of the active layer is crucial for the commercial viability of OPVs [44]. High-efficiency OPVs with 100-nm active layers have managed to surpass 20% efficiency [45–51]. However, when scaling up to thicker layers or larger modules, efficiency often declines due to constraints related to film thickness [52–56].

In OPV device upscaling, thick films are crucial. They boost light absorption by increasing the light path, enhancing photocurrent and efficiency, and ensuring performance as device size grows. During film preparation from dilute solutions, weak solution–substrate interactions that from the wettability and surface tension of the solution can lead to bubble formation and pinholes. Thick films can fill potential voids, improve film continuity and uniformity, and boost device reliability. They also enhance device robustness, making flexible OPV devices more resilient to bending or stretching stresses during handling and installation. Moreover, thick films are more fault-tolerant in large-scale production, mitigating the impact of deposition parameter fluctuations (like temperature and humidity), and improving manufacturing consistency and yield, which is crucial for OPV commercialization [42, 57–64].

An ideal 100-nm active layer enables the uniform distribution of donor (*D*) and acceptor (*A*) materials, optimal phase separation, and high purity, thereby enhancing the dynamics of excitons and charge carriers [65, 66]. The exciton diffusion length (*L*_D_), typically within 20 nm, is a primary limitation. As the active layer thickens, the domain size often increases, hindering exciton diffusion and resulting in greater exciton losses. However, a thick film also introduces complex trade-offs through increased carrier recombination issues and the challenges in maintaining morphological uniformity across large-area coatings.

The performance of OPVs with thicker active layers is unexpectedly degraded, primarily due to uneven light distribution, which limits the utilization of light energy. The limited diffusion and lifetime of excitons in thick films hinder their effective dissociation at the D/A interface [67]. Additionally, the increased migration distance for charge carriers after dissociation in thicker films impairs their transport [68, 69], while mismatches in mobility lead to space charge-carrier accumulation, increasing charge-carrier loss and reducing charge-carrier collection efficiency. Defects or impurities in thick films act as recombination centers, further elevating the probability of charge-carrier recombination [70–73]. Therefore, managing exciton and charge-carrier behavior is crucial for achieving high efficiency in thick-film OPVs.

This review focuses on the critical processes that influence photovoltaics performances in thick-film OPVs, including exciton generation, diffusion, dissociation, charge-carrier transport, and recombination. We evaluate recent advancements in material design and device engineering to identify key challenges and technologies essential for developing efficient thick-film OPVs, study the stability, cost and machine learning benefits of thick-film OPVs, aiming to support both theoretical understanding and practical knowledge for their commercialization.

## Mechanisms and Efficiency Challenges in Thick-Film OPVs

**Fig. 1:**
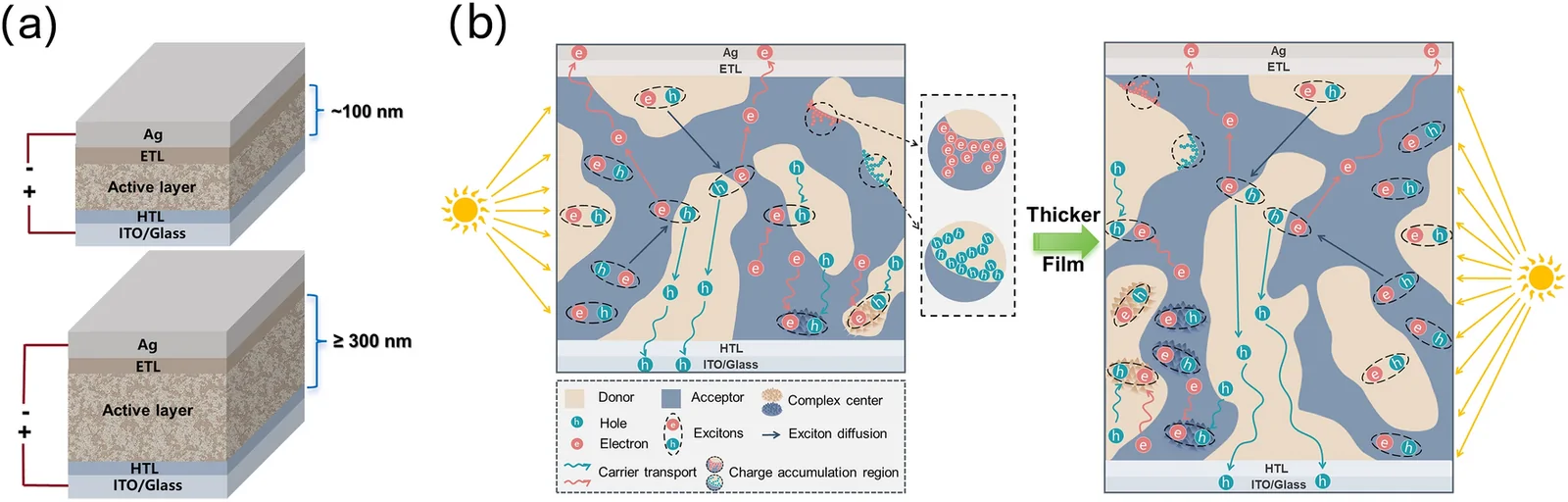
**a** Illustration for device structures of OPVs with thin-film and thick-film active layer. **b** Schematic illustration of exciton and charge-carrier behaviors in thin-film and thick-film devices

A traditional OPV device is structured in a sandwich configuration, consisting of five distinct layers as shown in Fig. 1a. The standard high-efficiency OPVs stack begins with an indium tin oxide (ITO)-coated glass substrate, which serves as the transparent anode. Next is the hole transport layer (HTL), which establishes an ohmic contact with the active layer and selectively conducts holes while blocking electrons. The active layer, the core component of the OPVs, contains the electron donor and acceptor materials responsible for photoelectric conversion. Adjacent to this is the electron transport layer (ETL), which facilitates electron transfer to the cathode, mirroring the HTL’s role for holes. The device is capped with a metallic cathode for charge-carrier collection [74–76]. While the thickness of active layer in typical high-efficiency OPVs is around 100 nm, those exceeding 300 nm are considered as thick-film OPVs. In addition, OPV devices have various structural types. Besides the typical bulk heterojunction structure mentioned above, there are also planar, bilayer, pseudo-bilayer, single-layer, and device structures without a transport layer. These different structural types have their own advantages and disadvantages. The choice of the appropriate structure should be based on specific application requirements and material properties (as discussed in the specific sections).

The quest for efficient spectral utilization and industrial scalability has driven increased research into thick-film OPVs. However, these devices face significant challenges due to the finite *L*_D_, reduced charge-carrier mobility (*μ*) and pronounced charge-carrier recombination. These factors fundamentally limit the photovoltaic performance of thick-film OPVs. Researchers have observed a marked decrease in PCEs with increasing active layer thickness in thick-film OPVs. This decline is linked to the thickness’s impact on exciton and charge-carrier behaviors. As depicted in Fig. 1b, sunlight penetrates the anode and is absorbed by the active layer. In thick-film devices, this provides a longer photon path, thereby enhancing exciton generation. However, the non-uniform light field distribution in these devices leads to uneven exciton generation and reduced spectral utilization [77].

Excitons generated in thick films must travel longer distances to reach the D/A interface for dissociation, a process limited by the exciton’s finite lifetime of approximately 5–20 nm. Consequently, many excitons are annihilated before reaching the interface, significantly reducing their effective utilization. After dissociation, free electrons and holes can transport along their respective pure phases and are conducted through their dedicated transport layers to the cathode and anode. However, the increased thickness can lead to space charge-carrier accumulation due to unbalanced charge-carrier accumulation and the relatively low charge-carrier mobility, resulting in the formation of high and low electric field regions that hinder efficient transport and collection.

Moreover, the higher incidence of morphological defects in thick active layers contribute to notable non-radiative recombination losses. Additionally, the variable stacking of donor and acceptor molecules might lead to disparities in charge-carrier mobilities. Despite their superior light absorption for better spectral utilization and manufacturing scalability, the performances of thick-film devices are hindered by constraints such as short exciton lifetime, low and unbalanced charge-carrier mobility, and significant recombination. Thus, we will discuss strategies and approaches to optimize the exciton and charge-carrier dynamics to improve the photovoltaic performances of thick-film OPVs and advancing their industrial application.

## Exciton and Charge-Carrier Behavior in Thick-Film Devices

### Exciton Generation and Diffusion: Improving Photon Utilization

**Fig. 2:**
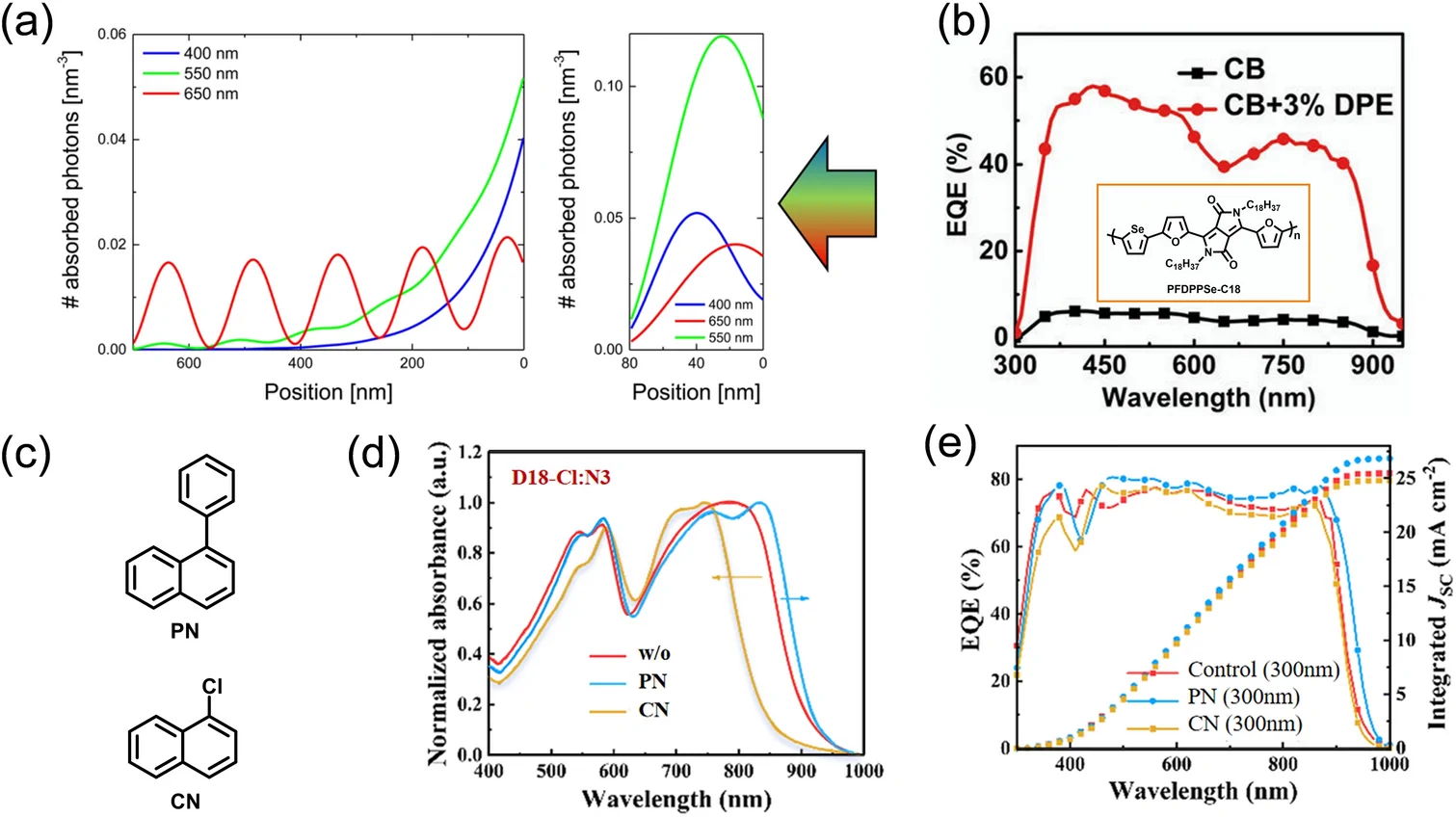
**a** Transfer matrix modeling calculates PCDTBT:PC_71_BM-based carrier generation profiles in thin (80 nm) and thick (700 nm) junctions under AM1.5G illumination. **a** is reprinted with permission from [81], copyright 2015 American Chemical Society. **b** EQE spectra of PFDPPSe-based devices and the chemical structure of PFDPPSe. **b** is reprinted with permission from [83], copyright 2018 The Royal Society of Chemistry. **c** Chemical structure of PN and CN. **d** Absorption spectra of D18-Cl:N3 films with various solvent additive. **e** EQE spectra of corresponding devices with 300 nm thickness. **d-e** are reprinted with permission from [85], copyright 2024 The Royal Society of Chemistry

Organic semiconductor materials generate excitons upon photoexcitation, and the enhancement of spectral utilization by the active layer is fundamental to the improvement of photocurrent [78]. Studies have revealed that increasing the film thickness does not effectively enhance the spectral utilization by the active layer [79, 80]. As depicted in Fig. 2a, Ardalan Armin et al. discovered that within a 700-nm-thick PCDTBT:PC_70_BM active layer, high-energy photons, such as blue and green light, are predominantly absorbed near the ITO surface [81]. In contrast, low-energy photons, like red light, penetrate deeper into the active layer, generating photocarriers throughout its volume. This results in a non-uniform distribution of photocarriers, which significantly impacts their transport and collection efficiency. This is attributed to the varying penetration depths of incident light of different wavelengths, with ultraviolet light having a shallow penetration depth and near-infrared light a deeper one. The non-uniformity of the thick-film active layer results in the ineffective utilization of light across different wavelengths.

To better harness the near-infrared region of sunlight, researchers have proposed using low-bandgap materials to enhance photon capture efficiency in OPVs [82]. For instance, a polymer (PFDPPSe) was synthesized by combining selenophenes with DPP-based acceptors (Fig. 2b), resulting in a maximum absorption wavelength of 830 nm and an absorption onset at 930 nm [83]. This approach successfully improved spectral utilization, achieving a PCE of 6.16% in 210-nm-thick OPVs.

The thickness tolerance (TT) parameter reported in the literature is applied to evaluate thick-film devices for its accuracy and fairness in quantifying thickness-variation sensitivity [84]. The calculation formula for TT is shown as follows:

1 $${\mathrm{TT}} = \frac{{{\mathrm{T}}@{\mathrm{PCE}}_{{{\mathrm{max}}}} }}{{\mathrm{S}}}$$

Here, T@PCE_max_ is the active layer thickness at which the highest PCE is achieved, measured in nanometre, and *S* is quantifies the sensitivity of PCE to thickness variation (the average efficiency loss per 1 nm increase in active layer thickness). The lower *S* indicates a stronger ability to maintain efficiency as thickness increases. The calculation formula is as follows:

2 $${\mathrm{S}} = \frac{{{\mathrm{PCE}}_{{{\mathrm{max}}}} - {\mathrm{PCE}}@{\mathrm{T}}_{{{\mathrm{max}}}} }}{{{\mathrm{T}}_{{{\mathrm{max}}}} - {\mathrm{T}}@{\mathrm{PCE}}_{{{\mathrm{max}}}} }}$$

Here, PCE_max_ is the highest PCE, PCE@*T*_max_ is the PCE at the maximum active layer thickness, and *T*_max_ is the maximum active layer thickness.

Moreover, in our analysis, we have also taken into account the influence of *T*_max_ on the TT value. Here, we introduced the concept of “relative thickness range (RTR)”:

3 $${\mathrm{RTR}} = \frac{{{\mathrm{T}}_{{{\mathrm{max}}}} - {\mathrm{T}}@{\mathrm{PCE}}_{{\text{max }}} }}{{{\mathrm{T}}_{{{\mathrm{max}}}} }}$$

By standardizing the thickness range relative to *T*_max_, RTR can eliminate deviations caused by different *T*_max_ values in different studies. To comprehensively assess materials, we suggest combining TT with PCE_max_. For example, a new parameter TT_RTR_ can be defined as follows:

4 $${\mathrm{TT}}_{{{\mathrm{RTR}}}} = \frac{{{\mathrm{PCE}}_{{{\mathrm{max}}}} }}{{{\text{S }} \times {\text{ RTR}}}}$$

In the following parts, the TT and TT_RTR_ values will be calculated and discussed for various thick-film systems.

Furthermore, thickness-insensitive OPVs were developed using 1-phenylnaphthalene (PN) as solvent additive (Fig. 2c) [85]. The active layer (D18-Cl:N3) treated with PN exhibited a redshift about 22 nm and broadening of absorption with a full width at half maximum (FWHM) of 256 nm (Fig. 2d, e), achieving a 16.48% PCE in a 300-nm-thick device, with a TT of 14,610 nm^2^ and TT_RTR_ of 3,510 nm^2^ (Table 1).

**Table 1:** Photovoltaic performances of OPVs with various film thickness and optimized *L*_D_ (discussed in this review)

Active Layer	Thickness (nm)	*V*_OC_ (V)	*J*_SC_ (mA·cm^−2^)	FF (%)	PCE (%)	TT (nm^2^)	TT_RTR_ (nm^2^)	Refs.
D18-Cl:N3	150	0.855	27.68	76.13	18.02	14,610	3510	[85]
	300	0.827	27.69	71.98	16.48			
PBDB-T:IDTT-OB	150	0.910	16.58	74.00	11.19	15,151	2826	[91]
	250	0.893	16.68	68.50	10.20			
PM6:BTP-eC9:L8-BO-F	120	0.852	27.26	79.80	18.53	13,734	2791	[92]
	300	0.836	28.36	73.00	17.31			
	500	0.835	27.49	66.40	15.21			
PM6:C6C4-4Cl:BTIC-4F	100	0.861	24.24	74.60	15.62	9236	2062	[93]
	330	0.835	23.61	66.30	13.07			
PM6:HD-1:BO-4Cl	100	0.848	28.56	80.00	19.42	10,362	3019	[94]
	300	0.831	29.75	70.82	17.49			
PM6 + PS:L8-BO	100	0.894	26.10	81.60	19.05	13,114	3123	[95]
	300	0.885	26.14	78.46	18.15			
	500	0.876	25.86	70.66	16.00			
PM6:L8-BO	110	0.887	26.85	80.10	19.10	11,305	2547	[96]
	300	0.873	26.61	73.90	17.20			
	480	0.863	24.80	72.20	15.50			
PTB7-Th:ITIC	101	0.823	14.45	64.32	7.65	6768	911	[97]
	133	0.827	14.25	61.78	7.28			
	165	0.809	14.29	59.29	6.85			
	196	0.811	13.11	58.65	6.26			
	231	0.824	12.82	53.65	5.71			
D18/BTP-eC9-4F	100	0.870	27.96	79.63	19.36	15,384	4408	[98]
	300	0.868	30.66	67.82	18.06			

**Fig. 3:**
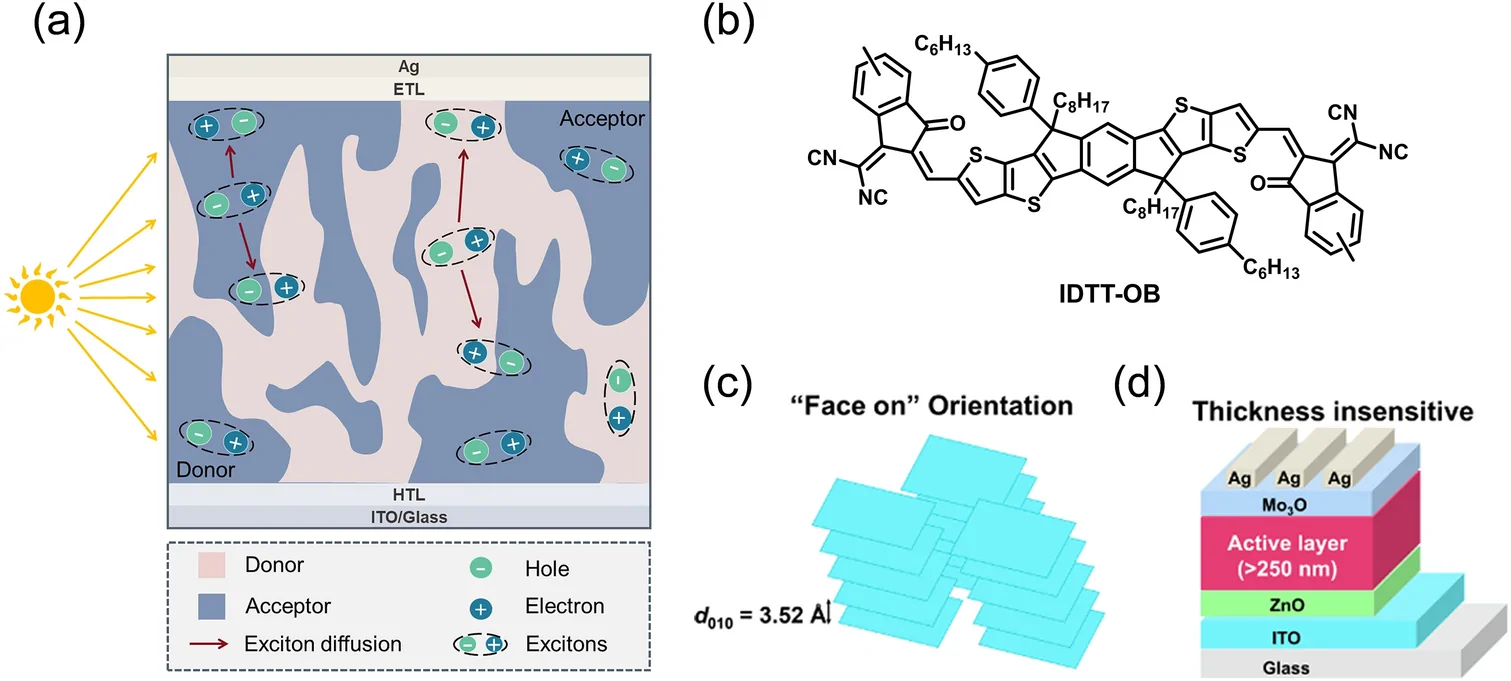
**a** Schematic illustration of exciton generation and diffusion. **b** Chemical structures of IDTT-OB. **c** Scheme of molecular packing mode with *π*-*π* stacking distances (*d*_010_) of 3.52 Å. **d** Thick-film device structure of IDTT-OB-based OPV. **c-d** are reprinted with permission from [91], copyright 2019 American Chemical Society

Exciton diffusion in OPVs active layers refers to the movement of photoexcited electron–hole pairs (excitons) within the material before reaching the D-A interface [86–88], as illustrated in Fig. 3a. The exciton diffusion length *L*_*D*_ is a parameter that describes the ability of excitons to diffuse within a material, defined as the average distance that an exciton can travel before recombining. The common formula for calculating the *L*_*D*_ is based on the three-dimensional exciton diffusion model:

5 $$L_{D} = \sqrt {D\tau }$$

wherein *D* represents the diffusion coefficient of exciton, which describes the rate at which exciton diffuse within the material. τ denotes the lifetime of exciton, referring to the average time from their generation to recombination. The exciton diffusion coefficient *D* can be calculated by the following formula:

6 $$D = \frac{\alpha }{8\pi R}$$

Here, *α* refers to the bimolecular exciton annihilation rate constant, which quantifies the rate at which two excitons meet and annihilate each other. *R* is the exciton annihilation radius, the distance within which excitons are likely to encounter and annihilate. In practical applications, the measurement of *L*_D_ can be achieved through techniques such as time-resolved photoluminescence (TRPL) or transient absorption (TA) spectroscopy. By analyzing the data from these experiments, one can determine the exciton lifetime and annihilation rate constant, which then allows for the calculation of the *L*_D_ [89, 90].

Usually, *D* is influenced by factors such as the material’s crystallinity and intermolecular interactions, while τ is affected by the degree of non-radiative recombination in the system. Consequently, the chemical structure and molecular packing of the material play a crucial role in determining the system's exciton* L*_*D*_. A longer exciton *L*_*D*_ is beneficial for effective charge-carrier generation in thicker active layers, which is essential for enhancing the short-circuit current density (*J*_SC_) and overall photovoltaic performance.

#### Non-Fullerene Acceptors for Enhanced Exciton Diffusion

Non-fullerene acceptors (NFAs) offer significant flexibility in adjusting the optical bandgap, energy levels, and molecular structure. By employing rational design of end groups, side-chain and central cores, the synthesis of new NFA materials with strong crystallinity and good intermolecular interactions can effectively increase the exciton* L*_D_ [99–105], enabling the construction of efficient thick-film devices.

Asymmetric structure typically exhibits stronger intermolecular interactions, which could help form tighter and more ordered molecular packing structures. For instance, the Bo’s group developed a seven-heterocyclic fused-ring acceptor, IDTT-OB (Fig. 3b), with asymmetric substituents in the side chain [91]. When combined with PBDB-T, the blend film exhibited more ordered molecular packing and enhanced crystallinity, with strong face-on orientation and ideal nanostructure with a domain size of 19 nm (Fig. 3c), which is beneficial for exciton diffusion (Table 2). Devices based on IDTT-OB showed insensitivity to the active layer thickness. Even with an active layer thickness of 250 nm (Fig. 3d), the average PCE of the device could still reach 10.20%, with a TT of 15,151 nm^2^ and TT_RTR_ of 2,826 nm^2^ (Table 1).

**Table 2:** Exciton *L*_D_ in various OPV systems

Active Layer	*L*_D_ (nm)	Refs.
BTP-C9	36.60	[92]
L8-BO-F	44.40	
BTP-C9:L8-BO-F	47.00	
C6C4-4Cl	9.03	[93]
BTIC-4F	7.95	
C6C4-4Cl:BTIC-4F	10.60	
PM6	10.96	[95]
PM6 + PS	13.79	
L8-BO	34.00	[96]
L8-BO (DIO)	37.00	
L8-BO (DICO)	45.00	
BTP-eC9-4F (CF)	26.94	[98]
BTP-eC9-4F (CF + OXY)	27.71	

**Fig. 4:**
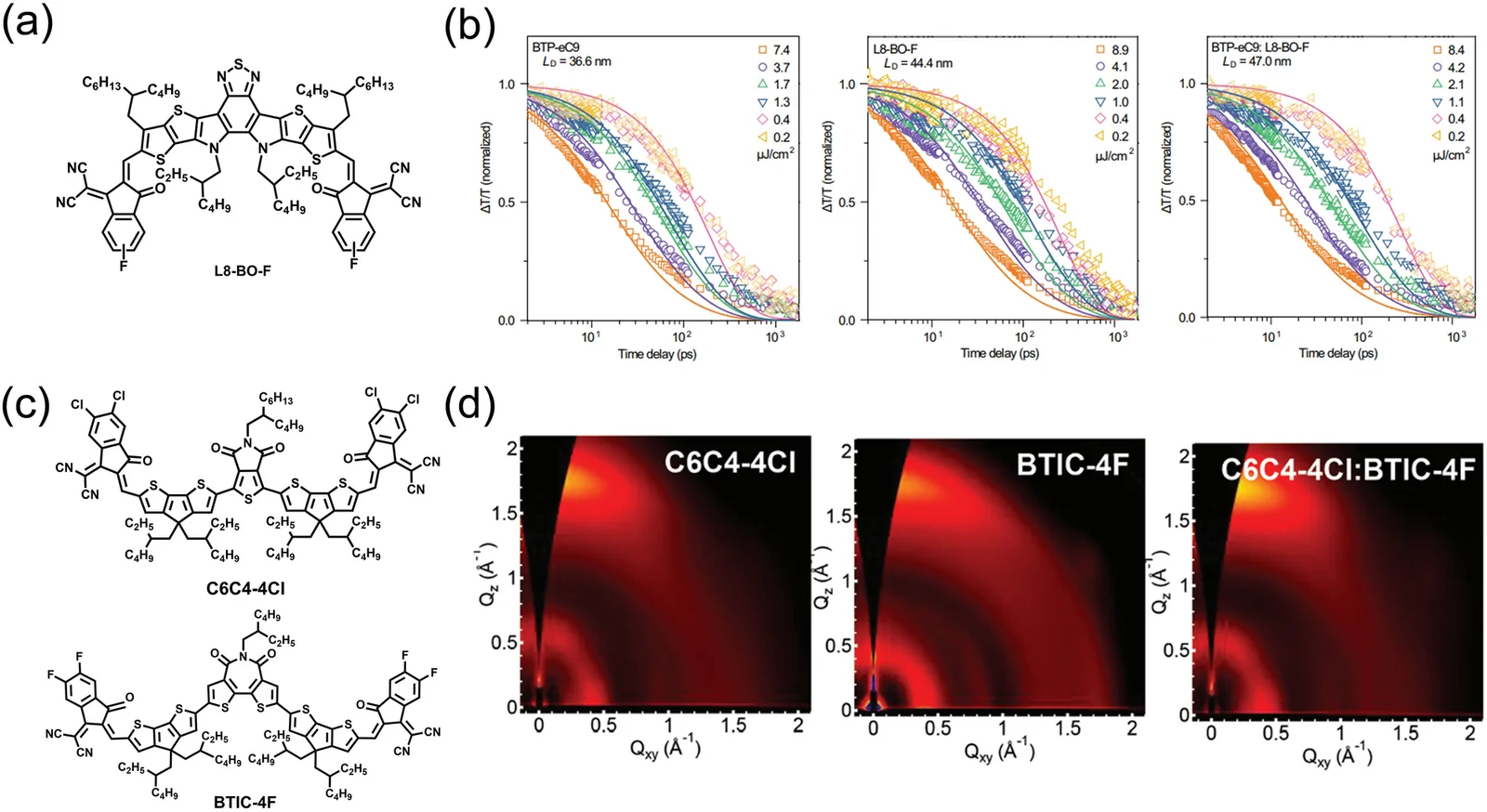
**a** Chemical structure of L8-BO-F. **b** The dynamics of the singlet excitons measured with the 670-nm pump excitation at different densities in films of BTP-eC9 (850 nm), L8-BO-F (814 nm) and BTP-eC9:L8-BO-F (850 nm). **b** is reprinted with permission from [92], copyright 2022 Nature Communication. **c** Chemical structure of C6C4-Cl and BTIC-4F. **d** GIWAXS patterns of C6C4-4Cl, BTIC-4F, and C6C4-4Cl:BTIC-4F blend films. **d** is reprinted with permission from [93], copyright 2023 Wiley–VCH

Sun's team introduced fluorinated material (L8-BO-F, in Fig. 4a, b) that improved exciton *L*_D_ (*L*_*D,* BTP-eC9_ = 36.6 nm, *L*_*D,* L8-BO-F_ = 44.4 nm, *L*_*D,* BTP-eC9:L8-BO-F_ = 47.0 nm, Table 2) and reduced recombination [92]. The 120-nm thin-film and 500-nm-thick exhibit the root-mean-square values (RMS) of 1.50 and 1.76 nm, respectively. The increased fibril width was observed in the thicker films. Using a layer-by-layer (LBL) deposition strategy, the devices based on PM6:BTP-eC9:L8-BO-F achieved certified PCEs of 17.31% at 300 nm and 15.21% at 500 nm, with a TT of 13,734 nm^2^ and TT_RTR_ of 2,791 nm^2^ (Table 1). Additionally, Aung Ko Kyaw’s group synthesized non-fused ring acceptors, C6C4-4Cl and BTIC-4F (Fig. 4c), which form uniform alloy-like phases. The film exhibits smoother and more uniform domains, with the RSM value decreasing from 2.78 to 1.78 nm [93]. This structure improved crystallinity (Fig. 4d) and exciton *L*_D_ (*L*_*D,* C6C4-4Cl_ = 9.03 nm, *L*_*D,* BTIC-4F_ = 7.95 nm, *L*_*D,* C6C4-4Cl:BTIC-4F_ = 10.6 nm, Table 2); by mixing these two acceptors with the donor PM6, ternary devices achieve PCEs of 15.62% at 100 nm and over 13% at 330 nm, with a TT of 9,236 nm^2^ and TT_RTR_ of 2,062 nm^2^ (Table 1), demonstrating the importance of phase uniformity. These studies highlight that molecular design strategies, such as asymmetric substitution, fluorination, and the formation of uniform alloy-like phases, are crucial for optimizing exciton dynamics, thereby enabling the construction of efficient thick-film OPVs.

#### High Crystallinity Small Molecule Donor

The electron donor also matters well for optimizing exciton diffusion. Recently, the Chen’s team introduced a high-crystallinity small-molecule donor, HD-1, which was incorporated into the PM6:BO-4Cl blend as a morphological regulator [94]. The introduction of HD-1 not only regulated the excessive aggregation of the acceptor in the high-boiling-point solvent CB but also achieved a longer exciton diffusion time (*τ*_2,_ obtained from the transient absorption spectrum analysis). The *τ*_2_ values for the binary and ternary OPVs were 3.07 and 4.66 ps, respectively. As a result, the device exhibited good tolerance to variations in the active layer thickness, achieving a PCE of over 17% with an active layer thickness of 300 nm, with a TT of 10,362 nm^2^ and TT_RTR_ of 3,019 nm^2^ (Table 1).

#### Controlling Molecular Packing

The spatial arrangement and stacking of donor and acceptor molecules within the active layer are crucial for facilitating efficient exciton diffusion. Ordered molecular alignment ensures continuous pathways for exciton diffusion and minimizes trap states, while optimal *π*-*π* stacking enhances intermolecular electronic coupling, reducing energy loss and improving exciton transport in thick-film OPVs.

**Fig. 5:**
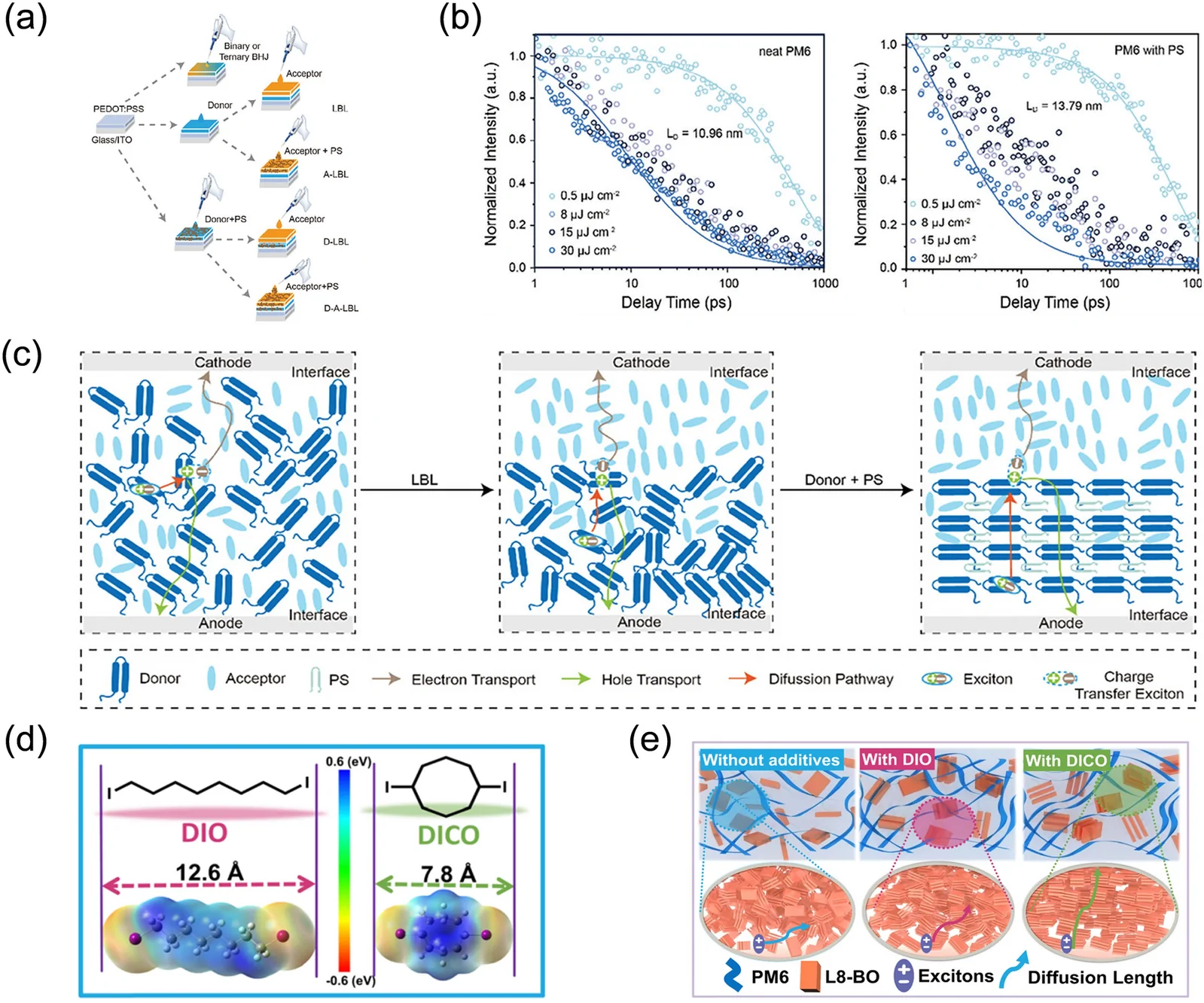
**a** Schematic diagram of different OPV device structures. **b** Pump fluence-dependent TA kinetics of PM6 and PM6 + PS films, traced at 600 nm. **c** Schematic illustration of the phase separation and excitons transport process. **a-c** are reprinted with permission from [95], copyright 2024 Wiley–VCH. **d** Chemical structure and calculated electrostatic potential (ESP) distribution of DIO and DICO. **e** Schematic diagram of the changes in the morphology of the PM6: L8-BO series blends before and after additive treatment. **d–e** are reprinted with permission from [96], copyright 2024 The Royal Society of Chemistry

Recently, the Hao’s research group successfully extended the exciton *L*_D_ in thick-film OPVs by incorporating insulating polymers, such as polystyrene (PS), into the active layer [95]. They utilized a molecular adsorption strategy combined with a LBL approach, as shown in Fig. 5a. This method effectively enhanced the *π*-*π* stacking between donor molecules, strengthening intermolecular interactions and the overlap of electronic wave functions. At the active layer thicknesses of 100 nm and 500 nm, the average correlation length (*ξ*) is 108.61 and 172.41 Å, respectively, indicating an increased donor-rich domain. thereby significantly increasing the exciton lifetime and *L*_D_ from 10.96 to 13.79 nm (Fig. 5b, c, Table 2). As a result, devices with the PM6 + PS:L8-BO blend achieved a PCE of 18.15% at 300 nm and 16.00% at 500 nm, with a TT of 13,114 nm^2^ and TT_RTR_ of 3,123 nm^2^ (Table 1). The Yang’s team synthesized a new halogenated alkane, 1,5-diiodocycloctane (DICO), and used it as a solvent additive in photovoltaic systems [96]. DICO exhibits a different electrostatic distribution (Fig. 5d), effectively induces ordered stacking of molecules in the active layer, and improves the phase separation size of donor and acceptor materials, thereby achieving longer exciton *L*_D_ is 45 nm in both donor and acceptor domains (Fig. 5e, Table 2). Devices based on PM6:L8-BO with DICO achieved PCEs of 17.20% at 300 nm and 15.50% at 480 nm, with a TT of 11,305 nm^2^ and TT_RTR_ of 2,547 nm^2^ (Table 1).

In addition, Ma’s team proposed in situ solvent annealing during spin coating (SC-SVA). It adjusts molecular packing in the active layer, improving non-fullerene acceptor crystallinity without significantly enlarging domain size (from 41.1 to 56.7 nm, enhancing device performance [97]. For instance, the PTB7-Th:ITIC-based device achieved 7.65% and 5.71% efficiency at 101 and 231 nm, with a TT of 6,768 nm^2^ and TT_RTR_ of 911 nm^2^ (Table 1). Bo et al. developed a mixed-solvent strategy to regulate acceptor molecule penetration into the donor layer of the LBL-treated OPV [98]. By mixing fast-evaporating chloroform (CF) with slow-evaporating o-xylene (OXY) and precisely adjusting their ratio, they finely tuned the spatial distribution of acceptor molecules in the active layer, and the crystal coherence length (CCL) increases from 27.96 to 29.43 Å for a 100-nm-thick active layer and from 21.51 to 24.31 Å for a 300-nm-thick active layer. This led to a vertically phase-separated structure and enhanced crystallinity of the acceptor phase, facilitating exciton diffusion (from 26.94 to 27.71 nm, Table 2). Consequently, the PCE of the D18/BTP-eC9-4F OPV was significantly improved, reaching 19.36% with a 100-nm-thick active layer. Impressively, when this strategy was applied to a 300-nm-thick active layer OPV, a PCE of 18.06% was achieved, with a TT of 15,384 nm^2^ and TT_RTR_ of 4,408 nm^2^ (Table 1).

The diffusion of excitons is closely related to domain size, which affects OPV performance. Research shows that a domain size of 10–20 nm is optimal for exciton diffusion to the D/A interface, promoting effective exciton separation. Even in systems with the same domain size, exciton lifetime and diffusion coefficient may differ, with longer diffusion lengths generally benefiting exciton dissociation efficiency. To make efficient OPV, balancing phase separation size and exciton diffusion importance. These demonstrate the significant potential of these novel additives, annealing process and different solvents in molecular packing control, and exciton *L*_D_ extending and efficient thick-film devices constructing, providing concrete ideas for commercial development.

### Exciton Dissociation: Key Factors for Free Charge-Carrier Generation

**Fig. 6:**
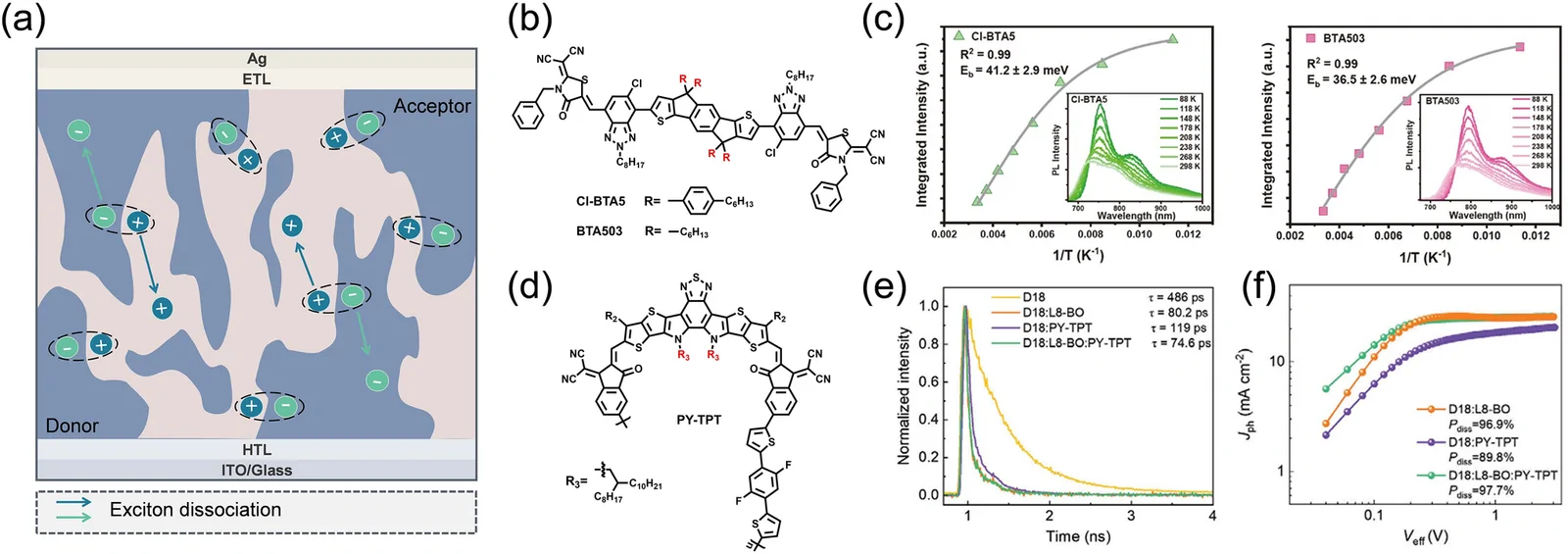
**a** Schematic illustration of exciton dissociation. **b** Chemical structures of BTA503. **c** Integrated PL intensity as a function of temperature (Inset: temperature-dependent PL spectra excited at 600 nm) of Cl-BTA5 and BTA503. **c** is reprinted with permission from [111], copyright 2024 Springer Nature. **d** Chemical structures of PY-TPT. **e** TRPL spectra of D18 and as-cast blend films. **f** *J*_ph_-*V*_eff_ curves of as-cast OPVs. **e–f** are reprinted with permission from [112], copyright 2024 Wiley–VCH

Exciton dissociation is a key process in OPVs, where photo-generated excitons at the D/A interface must overcome their Coulombic binding energy to separate into free charge carriers [106], as depicted in Fig. 6a. The efficiency of this dissociation process is influenced by several factors, including the exciton binding energy (*E*_b_), the phase separation at the D/A interface, and the number of available pathways for dissociation [107–110]. Optimizing these factors is crucial for enhancing the photocurrent and overall efficiency, especially in thick-film devices, where exciton diffusion distances are longer.

#### Reducing Exciton Binding Energy

Lower exciton *E*_b_ facilitates more efficient exciton dissociation. The Zhou’s group designed a wide bandgap NFA, as depicted in Fig. 6b. BTA503 (*E*_b_ = 36.5 meV) with reduced exciton *E*_b_ is designed by changing the phenyl side chain on the central core of Cl-BTA5 (*E*_b_ = 41.2 meV) to an alkyl chain [111]. BTA503 exhibits diverse *π*-*π* interactions, enhanced molecular stacking and appropriate domain size for phase separation (from the pure film of BTA503 to the blended film of PTQ10:BTA503, the RMS decreased from 5.93 to 1.21 nm), which are responsible for its reduced *E*_b_ (Fig. 6c). This small *E*_b_ leads to diminished charge recombination and fast exciton dissociation, thereby favoring the generation of more charge carriers in the active layer. Ultimately, the binary device based on PTQ10:BTA503 achieved a PCE of 10.13% at a film thickness of 306 nm, with a TT of 9,089 nm^2^ and TT_RTR_ of 1,512 nm^2^ (Table 3). *E*_b_ is also related to the relative dielectric constant (*ε*_r_) of the material; increasing *ε*_r_ will reduce the *E*_b_ of CT excitons [113]. Huang et al. enhanced an as-cast OPV by adding a high-dielectric-constant polymer acceptor PY-TPT (Fig. 6d) to the D18:L8-BO blend, creating a double-fibril network; the RMS increases from 1.26 to 1.31 nm [112]. This improved the active layer’s dielectric constant and lowered its exciton *E*_b_ (increasing *ε*_r_ will reduce the *E*_*b*_ of CT excitons, *ε*_r, D18:L8-BO_ = 4.17, *ε*_r, D18:L8-BO:PY-TPT_ = 4.82) enabling efficient exciton dissociation and charge transport (Fig. 6e, f). The D18:L8-BO:PY-TPT device thus reached a record PCE of 17.54% at 300 nm thickness, with a TT of 18,867 nm^2^ and TT_RTR_ of 5,264 nm^2^ (Table 3).

**Table 3:** Photovoltaic performances of the thick-film OPVs with various thickness and high exciton dissociation efficiency (discussed in this review)

Active Layer	Thickness (nm)	*V*_OC_ (V)	*J*_SC_ (mA·cm^−2^)	FF (%)	PCE (%)	TT (nm^2^)	TT_RTR_ (nm^2^)	Refs.
PTQ10:BTA503	160	1.112	15.86	72.03	12.70	9089	1512	[112]
	306	1.102	17.30	55.00	10.13			
D18:L8-BO:PY-TPT	100	0.926	25.67	78.27	18.60	18,867	5264	[113]
	300	0.901	26.26	74.13	17.54			
PM6:P(BTzE-BDT):BTP-eC9	100	0.870	28.30	81.20	20.0	11,111	3333	[115]
	300	0.830	28.40	76.70	18.20			
D18/L8-BO	110	0.917	26.80	78.30	19.20	13,406	3000	[116]
	500	0.886	28.00	64.90	16.00			
PM1/L8-BO	50	0.900	27.08	77.16	18.81	1940	1011	[117]
	180	0.900	24.91	68.97	15.46			

#### Balancing Phase Separation Size and Purity

In addition to reducing exciton *E*_b_, optimizing the phase separation between donor and acceptor materials is equally critical for enhancing exciton dissociation. In thin-film OPVs, the domain size is usually small. This is because the thin film thickness of 80–120 nm limits the scale of phase separation, making the mixing of donor and acceptor materials more uniform. In contrast, in thick-film OPVs, the domain size is typically larger and excitons is difficulty diffusing. This is because the thicker film provides more space, allowing donor and acceptor materials to form larger phase-separated regions. Therefore, obtaining good phase separation size and purity in thick-film OPVs is crucial. A well-balanced phase separation can ensure a sufficient number of interfaces for efficient exciton dissociation while minimizing recombination losses.

The design and optimization of donors are also conducive to efficient exciton dissociation. Shi and colleagues introduced a novel wide band-gap polymer donor, P(BTzE-BDT), into the PM6:BTP-eC9 binary system [114]. This not only broadens the absorption spectrum and enhances photon harvesting but also promotes dense molecular packing and reduces domain size. The RMS roughness values of the binary and ternary devices are 1.20 nm and 0.91 nm, respectively. The photoluminescence (PL) quenching efficiency of the ternary system reaches 98.4%, higher than the binary system’s 89.5%, indicating that P(BTzE-BDT) effectively reduces exciton recombination loss. The increased D/A interface also facilitates exciton dissociation. As a result, a PCE of 18.2% is realized with an active layer thickness of 300 nm, with a TT of 11,111 nm^2^ and TT_RTR_ of 3333 nm^2^ (Table 3).

**Fig. 7:**
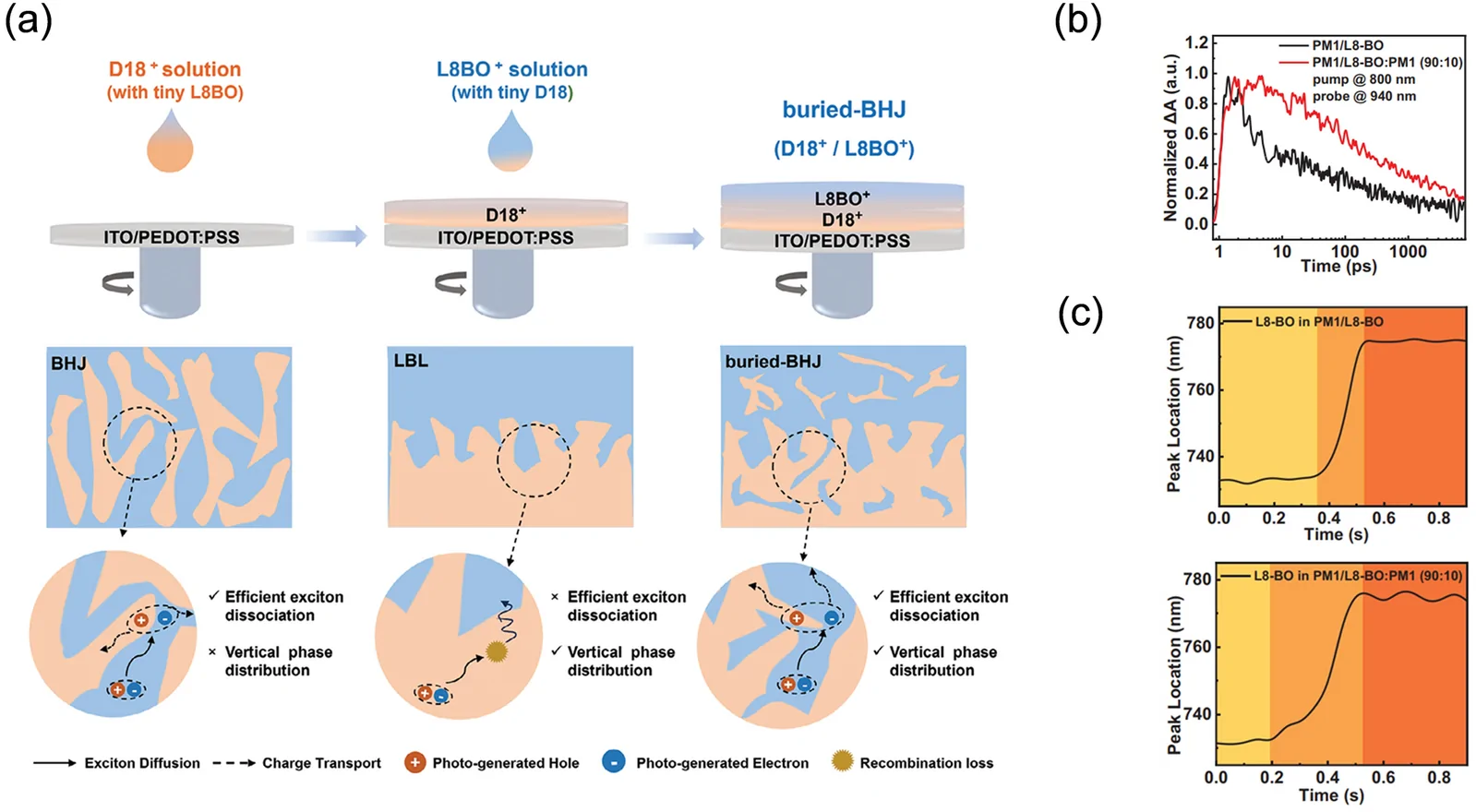
**a** Diagram of fabrication procedures of buried-BHJ structures, and the schematic illustration of vertical phase distribution in BHJ, LBL, and buried BHJ films and corresponding exciton/charge dissociation and recombination behaviors. **a** is reprinted with permission from [115], copyright 2024 Wiley–VCH. **b** TA kinetic curves probed at 940 nm for PM1/L8-BO and PM1/L8-BO:PM1 (90:10, wt/wt) films under 800-nm pump with power fluxes of 20 μJ cm^−2^. **c** The peak location of L8-BO dependence on L8-BO layer formation time. **b–c** are reprinted with permission from [116], copyright 2024 Wiley–VCH

The Bo’s team introduced a novel sequentially processed bulk-heterojunction buried structure (buried-BHJ) for efficient thick-film OPVs [115]. This structure achieves a rational distribution of donor and acceptor phases in the vertical direction and embeds a large number of D/A interfaces, promoting exciton dissociation. In addition, the buried-BHJ film exhibits a *π*-*π* stacking peak at *q*_*z*_ = 1.58 Å^−1^ with a CCL of 25.12 Å, which is significantly higher than that of the BHJ film. This indicates that buried-BHJ film has a more appropriate domain structure size, which can maintain rapid charge-carrier transport capabilities, significantly reducing recombination losses, as demonstrated in Fig. 7a. The buried-BHJ structure based on D18/L8-BO achieved a PCE of 16.0% on a 500-nm-thick active layer, with a TT of 13,406 nm^2^ and TT_RTR_ of 3,000 nm^2^ (Table 3), demonstrating its potential and efficiency advantages in large-scale manufacturing. The Zhang’s research group improved exciton utilization near the cathode by incorporating a small amount of donor into the acceptor layer (DIA strategy) combined with a LBL deposition method, achieving a reasonable phase distribution and generating a richer D/A interface (Fig. 7b, c). The CCL values associated with lamellar stacking in the IP direction and π-π stacking in the OOP direction can be concurrently increased from 50.22 to 52.75 Å and from 18.87 to 22.35 Å by incorporating 10 wt% PM1 into L8-BO layer, ensuring suitable phase separation size and effective exciton dissociation [116].With the DIA strategy, the PCE of the binary system PM1/L8-BO increased from 18.02% to 18.81% at 50 nm and maintained 74.0% and 82.2% of the original value at 180 nm, with a TT of 1,940 nm^2^ and TT_RTR_ of 1,011 nm^2^ (Table 3), respectively.

These examples demonstrate that balancing phase separation size and purity effectively promotes exciton dissociation, improving the efficiency and scalability of thick-film OPVs.

### Charge-Carrier Transport: Enhancing Mobility and Balancing Dynamics

Charge-carrier mobility (*μ*) is an important physical quantity that describes the transport performance of chargecarriers (including electrons and holes) in semiconductor materials. In OPVs systems, the space-charge-limited current (SCLC) method is used to measure carrier mobility. Under high electric fields, the injection rate of carriers exceeds the recombination rate, forming a space-charge region [117]. The relationship between current and voltage can be used to calculate the carrier mobility. This method is based on the Mott–Gurney law and is applicable to the injection and transport of carriers under high electric fields. Under the SCLC effect, the relationship between *J* and *V* can be expressed as:

7 $$J = \frac{9}{8}\varepsilon_{r} \varepsilon_{0} \mu \frac{{V^{2} }}{{L^{3} }}$$

where *J* is the current density, *V* is the applied voltage, *L* is the thickness of the device, *ε*_r_ is the relative dielectric constant of the material, *ε*_0_ is the dielectric constant of free space, and *μ* is the charge-carrier mobility [118, 119]. It can be seen that the *J* decreases with an increase in the device’s thickness *L*, following the relationship *J* ∝ *L*^−3^. The thicker the device, the longer the distance that charge carriers need to traverse, thus maybe resulting in a decrease in current density [120, 121].

**Fig. 8:**
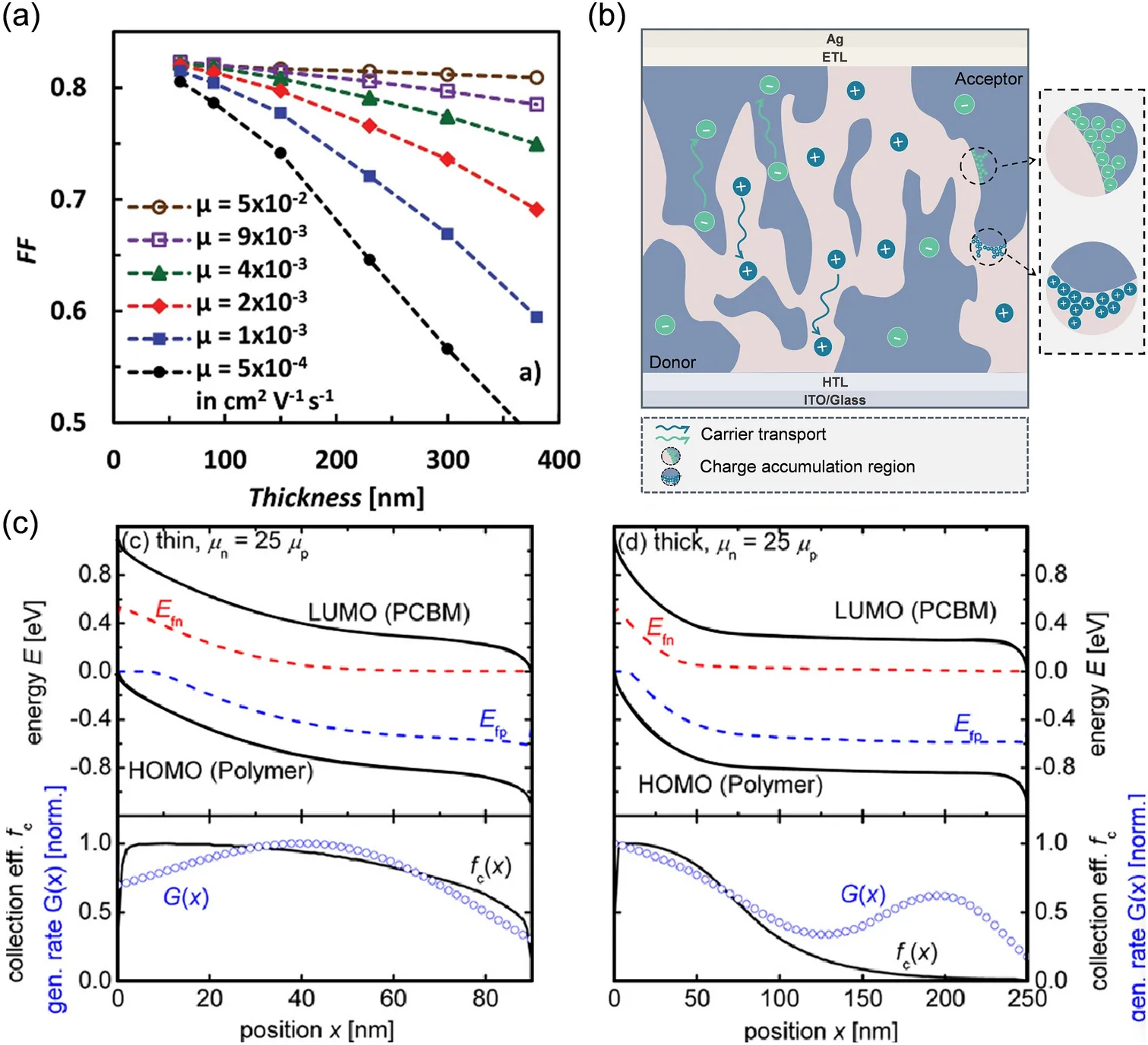
**a** FF as a function of active layer thickness for simulated P3HT:PCBM device with *µ*_e_ = *µ*_h_ and *k* = 2 × 10^–12^ cm^3^ s^−1^. **a** is reprinted with permission from [122], copyright 2015 Wiley–VCH. **b** Schematic illustration of charge-carrier transport and space-charge region. **c** Band diagrams of a 90-nm thin-film and a 250-nm thick-film OPV with asymmetric mobilities (*μ*_n_ ≫ *μ*_p_) at short-circuit under one sun illumination. The collection efficiency is high only in the depletion region, indicating reduced carrier collection near the front contact for the thick device. **c** is reprinted with permission from [132], copyright 2012 American Chemical Society

McGehee and colleagues examined the impact of charge-carrier mobility in thick-film OPVs using numerical one-dimensional drift–diffusion device simulators, which are typically used for inorganic solar cells [122]. In their study of the P3HT:PC_61_BM system, where the difference between hole and electron mobilities is negligible, devices with higher annealing temperatures, leading to higher mobilities, exhibited better FF (Fig. 8a). This highlights the critical role of mobility in reducing recombination rates and minimizing space-charge buildup [123]. The FF dependence on thickness diminished as charge-carrier mobility increased, demonstrating the importance of high mobility for efficient charge-carrier transport in thick-film OPVs [124–131].

As shown in Fig. 8b, charge-carrier transport balance refers to the dynamic equilibrium maintained between electron mobility (*μ*_e_) and hole mobility (*μ*_h_) during the transport process. If the balance is disrupted, the space-charge accumulation region could be formed [133, 134]. Charges in the low-field region cannot be collected in time, leading to severe non-radiative recombination, which directly affects the *J*_SC_ and FF of thick-film devices. Nelson et al. observed that in PCPDTBT:PC_71_BM thin- and thick-film devices, unbalanced mobilities lead to variations in charge-carrier collection efficiency [132]. In thick-film devices, as displayed in Fig. 8c, carriers must diffuse to the depletion region, which increases the likelihood of recombination. Their simulations indicated that devices with unbalanced mobilities show a significant decrease in* J*_SC_ as thickness increases, and FF is also affected.

Efforts to enhance charge-carrier mobility in BHJ devices have led to the development of high-performance thick-film OPVs. For example, a well-defined face-on orientation with strong *π*-*π* stacking in the *q*_z_-direction can result in high charge-carrier mobilities in both neat and blend films by establishing a vertical charge-carrier transport channel. Additionally, introducing highly crystalline ternary components or carefully controlling the morphology (such as forming nanofibers or improving vertical phase separation) can further enhance charge-carrier transport. These strategies will be discussed in more detail in the following sections.

#### Functionalized Acceptors for Efficient Charge Transport

Enhancing charge-carrier mobility in thick-film OPVs often involves optimizing the properties of acceptor materials [135–140]. One effective approach is the functionalization of acceptor molecules, which improves their molecular packing, crystallinity, and overall charge transport properties [141].

**Fig. 9:**
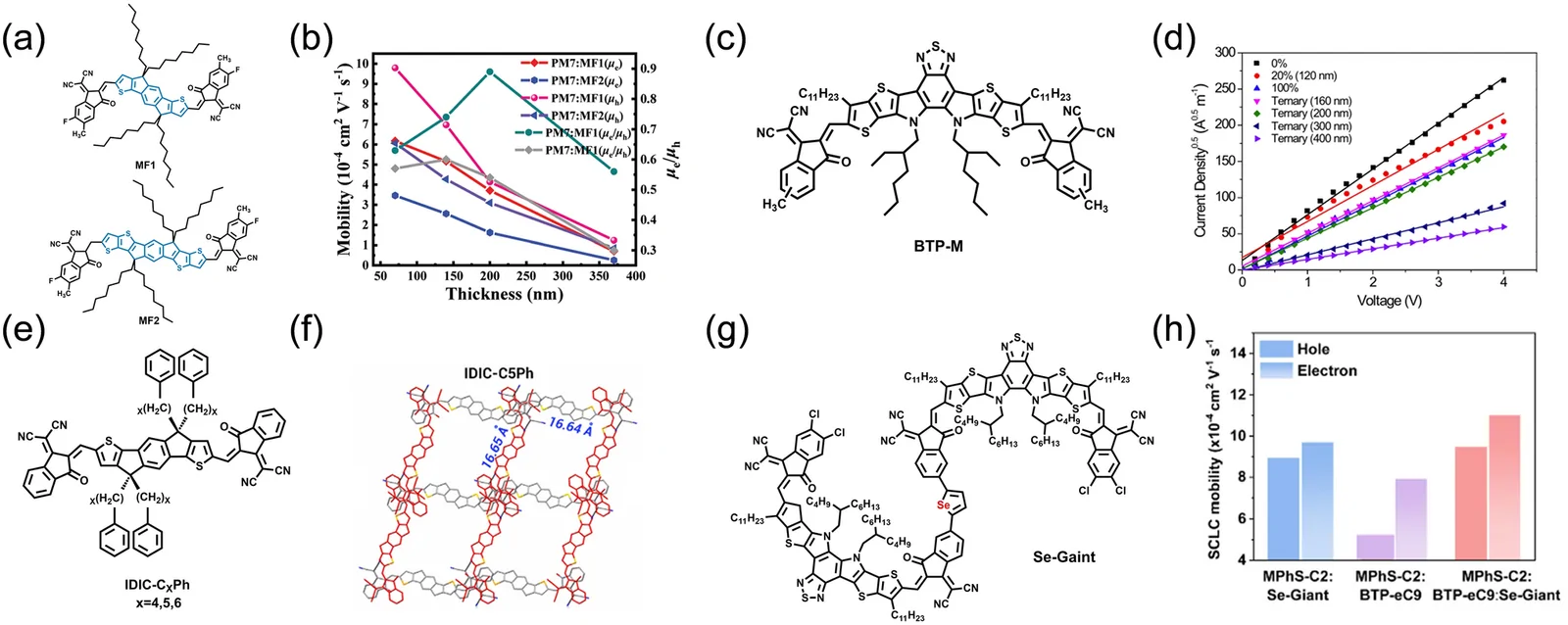
**a** Chemical structure of MF1 and MF2. **b** Changes of *µ*_e_, *µ*_h_, and *µ*_e_/*µ*_h_ under different thickness of active layer. **b** is reprinted with permission from [142], copyright 2020 Wiley–VCH. **c** Chemical structure of BTP-M. **d** *J*^0.5^-*V* curves of the electron-only devices based on PM6:Y6:BTP-M films with different weight ratios of BTP-M and ternary blends with various thickness. **d** is reprinted with permission from [143], copyright 2021 Wiley–VCH. **e** Chemical structure of IDIC-CxPh. **f** Molecular packing of IDIC-C5Ph in single crystal. **f** is reprinted with permission from [144], copyright 2021 Elsevier B.V. **g** Chemical structures of Se-Giant. **h** Histograms of *m*_e_ and *m*_h_ for the investigated binary and ternary systems. **h** is reprinted with permission from [145], copyright 2024 The Royal Society of Chemistry

In Yang’s study, two small-molecule acceptors MF1 and MF2 (Fig. 9a) were designed with fluorine and methyl dual-functional end groups, combined with alkyl-substituted fused aromatic cores, achieving ordered π-π stacking, high charge-carrier mobility (*μ*_e, PM7:MF1_ = 5.16 × 10^–4^ cm^2^ V^−1^ s^−1^, *μ*_h, PM7:MF1_ = 6.97 × 10^–4^ cm^2^ V^−1^ s^−1^, *μ*_e, PM7:MF2_ = 2.56 × 10^–4^ cm^2^ V^−1^ s^−1^, *μ*_h, PM7:MF2_ = 4.27 × 10^–4^ cm^2^ V^−1^ s^−1^) and transport balance (*μ*_h_/*μ*_e, PM7:MF1_ = 1.35, *μ*_h_/*μ*_e, PM7:MF2_ = 1.66, Fig. 9b) [142]. The FF decreased slowly with increasing thickness for MF1, demonstrating moderate thickness insensitivity, and thus, devices based on PM7:MF1 maintained efficiencies above 11% at thicknesses over 400 nm and above 10% at 500 nm, with a TT of 22,231 nm^2^ and TT_RTR_ of 2,714 nm^2^ (Table 4), offering insights into the design and optimization of thick-film materials. Chen’s group reported that a ternary strategy with high efficiency and thickness tolerance are developed using an alloy-like composite of Y6 and a newly designed derivative (Fig. 9c), BTP-M [143]. The BTP-M, which has higher energy levels and lower crystallinity than Y6 due to its electron-pushing methyl substituent, effectively optimizes the energy levels and morphologies of the active layers. Additionally, thick films can achieve higher charge transport properties compared to thin films, particularly in terms of *μ*_e_ (4.52 × 10^–3^ cm^2^ V^−1^ s^−1^) (Fig. 9d, Tables 4 and 5). These optimizations enable the device (PM6:Y6:BTP-M) to maintain excellent performance even with a thick active layer (300 nm), achieving an efficiency of 14.23%, with a TT of 9,464 nm^2^ and TT_RTR_ of 1,919 nm^2^ (Table 4), and highlighting its potential for practical applications. Bao’s team designed three acceptor molecules, IDIC-CxPh (*x* = 4, 5, 6), depicted in Fig. 9e, by controlling the number of flexible alkyl carbons [144]. IDIC-C_5_Ph exhibited a unique network-like structure, providing efficient dual charge-carrier transport channels: *μ*_e_ is 6.26 × 10^–4^ cm^2^ V^−1^ s^−1^, *μ*_h_ is 5.42 × 10^–4^ cm^2^ V^−1^ s^−1^ (Fig. 9f, Tables 4 and 5), and thus, devices based on PBDB-TF:IDIC-C_5_Ph achieving a maximum PCE of 14.56% and an FF of 80.02% at 110 nm and at 470 nm, the FF remained above 70%, with a PCE of 13%, with a TT of 26,338 nm^2^ and TT_RTR_ of 4,415 nm^2^ (Table 4).

**Table 4:** Photovoltaic performances of OPVs with various film thickness and with high & balance charge-carrier mobility (discussed in this review)

Active Layer	Thickness (nm)	*V*_OC_ (V)	*J*_SC_ (mA_·_cm^− 2^)	FF (%)	PCE (%)	TT (nm^2^)	TT_RTR_ (nm^2^)	*μ*_e_ (cm^2^ V^− 1^ s^− 1^)	*μ*_h_ (cm^2^ V^− 1^ s^− 1^)	*μ*_h_ /*μ*_e_	Refs
PM7:MF1	140	0.944	16.80	78.30	12.40	22,231	2714	5.16 × 10^− 4^	6.97 × 10^− 4^	1.35	[142]
	445	0.931	17.09	69.90	11.11			/	/	/	
	510	0.929	16.97	63.90	10.07			/	/	/	
PM7:MF2	140	0.957	19.20	74.50	13.70	13,770	1872	2.56 × 10^− 4^	4.27 × 10^− 4^	1.66	[142]
	438	0.955	19.35	59.80	11.04			/	/	/	
	500	0.953	19.20	54.90	10.04			/	/	/	
PM6:Y6:BTP-M	120	0.875	26.56	73.46	17.03	9464	1919	1.35 × 10^− 3^	3.38 × 10^− 3^	2.50	[143]
	300	0.855	26.87	62.06	14.23			4.52 × 10^− 3^	2.71 × 10^− 3^	0.60	
PBDB-TF:IDIC-C_5_Ph	115	0.948	19.19	80.02	14.56	26,338	4415	6.26 × 10^− 4^	5.42 × 10^− 4^	0.87	[144]
	470	0.921	20.15	70.12	13.01			/	/	/	
MPhS-C2:BTP-eC9:Se-Giant	125	0.903	26.34	76.33	18.16	19,565	4816	9.10 × 10^− 4^	9.46 × 10^− 4^	1.04	[145]
	305	0.892	26.50	71.96	17.01			/	/	/	
PTQ10:IDIC	130	0.969	17.81	73.60	12.70	9790	1647	6.72 × 10^− 4^	5.04 × 10^− 4^	0.75	[146]
	310	0.943	19.16	57.10	10.31			/	/	/	
PM6:PTQ10:PY-IT	138	0.940	23.79	73.87	16.52	8882	1937	6.81 × 10^− 4^	7.86 × 10^− 4^	1.15	[147]
	306	0.919	23.59	64.17	13.91			/	/	/	
P4T2F-HD:Y6-BO	110	0.720	24.39	75.30	13.56	17,863	3500	/	/	/	[148]
	300	0.710	25.92	67.70	12.48			/	/	/	
D18:ZW1:Y6	120	0.860	27.97	76.90	18.50	15,510	3146	5.15 × 10^− 4^	5.27 × 10^− 4^	1.02	[149]
	300	0.831	27.52	72.9	16.67			3.26 × 10^− 4^	4.15 × 10^− 4^	1.27	
PM6:L8-BO	100	0.880	26.68	80.50	19.02	5813	1843	7.12 × 10^− 4^	7.45 × 10^− 4^	1.05	[154]
	250	0.880	27.30	69.00	16.44			/	/	/	
PM6:BTP-eC9:EH-C_8_F_17_	100	0.836	27.30	79.20	18.03	21,929	5536	7.91 × 10^− 4^	9.79 × 10^− 4^	1.24	[155]
	350	0.822	28.70	71.50	16.89			/	/	/	
PM6:BTP-eC9	110	0.846	27.47	80.49	19.01	17,821	4248	/	/	/	[156]
	250	0.846	27.57	76.53	18.19			6.15 × 10^− 4^	8.06 × 10^− 4^	1.36	
	400	0.846	27.41	73.02	17.22			/	/	/	
PM6:PM6-PA:L8-BO	100	0.880	27.00	80.80	19.30	9523	2757	6.10 × 10^− 4^	5.40 × 10^− 4^	0.89	[162]
	300	0.850	28.00	72.10	17.20			/	/	/	
PM6:L8-BO	100	0.900	26.30	80.70	19.10	15,384	4408	1.34 × 10^− 4^	1.52 × 10^− 4^	1.03	[163]
	300	0.900	26.50	74.50	17.80			/	/	/

**Table 5:** Charge-carrier mobility in various OPV systems

Active Layer	*μ*_e_ (cm^2^ V^−1^ s^−1^)	*μ*_h_ (cm^2^ V^−1^ s^−1^)	*μ*_h_* /μ*_e_	Refs.
PM6:Y6	1.83 × 10^–3^	2.29 × 10^–3^	1.25	[143]
PBDB-TF:IDIC-C_4_Ph	2.47 × 10^–4^	3.20 × 10^–4^	1.30	[144]
PBDB-TF:IDIC-C_6_Ph	2.34 × 10^–4^	3.52 × 10^–4^	1.50	
MPhS-C2:Se-Giant	6.58 × 10^–4^	8.92 × 10^–4^	1.36	[145]
MPhS-C2:BTP-eC9	9.29 × 10^–4^	5.23 × 10^–4^	0.56	
PTQ10:IDIC (As-cast)	3.43 × 10^–4^	0.36 × 10^–4^	0.10	[146]
PTQ10:IDIC (TA)	4.80 × 10^–4^	3.21 × 10^–4^	0.66	
PTQ10:PY-IT	5.21 × 10^–4^	3.24 × 10^–4^	0.62	[147]
PM6:PY-IT	5.73 × 10^–4^	7.86 × 10^–4^	1.28	
D18:Y6 120 nm	3.12 × 10^–4^	3.65 × 10^–4^	1.16	[149]
D18:Y6 300 nm	2.31 × 10^–4^	2.31 × 10^–4^	1.00	
PM6:L8-BO (BC-type)	3.62 × 10^–4^	5.22 × 10^–4^	1.44	[154]
PM6:L8-BO (LBL-type)	5.09 × 10^–4^	6.72 × 10^–4^	1.32	
PM6:BTP-eC9	6.84 × 10^–4^	9.22 × 10^–4^	1.35	[155]
PM6:BTP-eC9 (BHJ)	2.15 × 10^–4^	7.13 × 10^–4^	3.31	[156]
PM6:BTP-eC9 (LBL)	5.16 × 10^–4^	7.19 × 10^–4^	1.39	
PM6-PA:L8-BO	8.50 × 10^–4^	7.90 × 10^–4^	0.93	[162]
PM6:L8-BO	5.30 × 10^–4^	4.00 × 10^–4^	0.75	
PM6:L8-BO	3.68 × 10^–5^	1.46 × 10^–5^	2.03	[163]

Min's group designed a giant molecule acceptor, Se-Giant, using the *Y*-series acceptor as the backbone and connecting it with selenophene, as depicted in Fig. 9g, improving charge-carrier mobility and balance: *μ*_e_ is 9.10 × 10^–4^ cm^2^ V^−1^ s^−1^, *μ*_h_ is 9.46 × 10^–4^ cm^2^ V^−1^ s^−1^ and *μ*_h_/*μ*_e_ is 1.04 (Fig. 9h, Tables 4 and 5) [145]. Incorporating Se-Giant into the MPhS-C2:BTP-eC9 system formed an alloy-like acceptor phase, optimizing charge-carrier transport channels. This all-small-molecule OPV system achieved a PCE of 18.16%, one of the highest efficiencies among similar works. Even at 300 nm, the system obtained a significant PCE of over 17.0%, with a TT of 19,565 nm^2^ and TT_RTR_ of 4,816 nm^2^ (Table 4).

#### Molecular Planarity (Donor Aspect)

Molecular planarity plays a key role in molecular packing and crystallinity. More planar molecules have higher crystallinity and are prone to form ordered packing, which is crucial for charge transport. In addition, molecular planarity also affects its compatibility with other components. Therefore, regulating molecular planarity has become an effective way to optimize the morphology of the active layer.

**Fig. 10:**
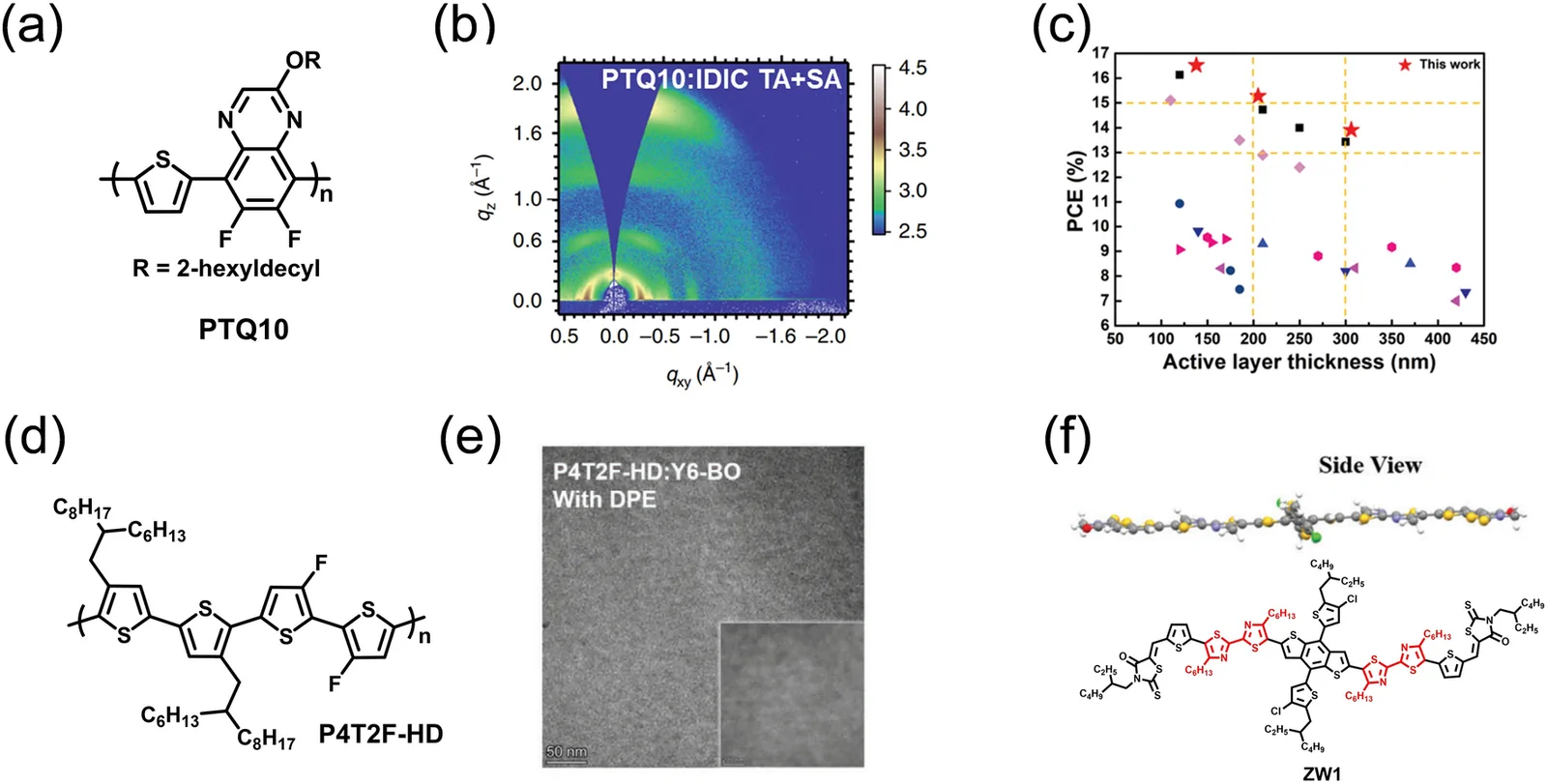
**a** Chemical structure of PTQ10.** b** GIWAXS images of PTQ10:IDIC films with TA + SA treatment.** b** is reprinted with permission from [146], copyright 2018 Springer Nature. **c** Plot of the PCE values versus photoactive layer thickness of the high-efficiency all-PSCs with thick photoactive layer reported in the literature. **c** is reprinted with permission from [147], copyright 2022 Wiley‐VCH. **d** Chemical structure of P4T2F-HD. **e** TEM images of the the P4T2F-HD:Y6-BO blend films with DPE additive. **d** is reprinted with permission from [148], copyright 2021 Wiley‐VCH. **e** Chemical structure and side view of the optimized geometry of ZW1. **e** is reprinted with permission from [149], copyright 2023 Wiley‐VCH

The Li’s group developed PTQ10 (Fig. 10a), a low-cost D-A copolymer donor. It uses thiophene as the donor unit and fluorinated quinoxaline as the acceptor unit [146]. Its alkoxy side chains enhance solubility and light absorption, boost molecular planarity by reducing steric hindrance, and the fluorine atoms lower the HOMO level to − 5.54 eV, improving crystallinity (Fig. 10b) and hole mobility. In thick-film devices, PTQ10 shows excellent performance. At 130-nm active layer thickness, the PCE reaches 12.70%, and even at 310 nm, it remains at 10.31%, with a TT of 9,790 nm^2^ and TT_RTR_ of 1647 nm^2^ (Table 4). After TA + SA treatment, the hole and electron mobilities of PTQ10:IDIC blends reach 5.04 × 10^−4^ and 6.72 × 10^−4^ cm^2^ V^−1^ s^−1^, respectively (Table 4). This balanced charge mobility cuts recombination and greatly ups the FF and *J*_SC_, proving PTQ10’s edge in thick-film devices and commercial potential. Then, Sun et al. introduced PTQ10 into the PM6:PY-IT system to optimize the active layer's molecular packing [147]. This reduced the $\pi - \pi$ stacking distance and increased the CCL value (21 Å for host binary and 25 Å for ternary blend), enhancing molecular planarity and order. As a result, the *μ*_h_ reached 7.86 × 10^−4^ cm^2^ V^−1^ s^−1^, and the *μ*_e_ reached 6.81 × 10^−4^ cm^2^ V^−1^ s^−1^ (Tables 4 and 5). Thus, the devices exhibited high thickness tolerance between 70 and 306 nm (Fig. 10c), delivering a PCE of 16.52% at 138 nm and retaining 13.91% at 306 nm (TT is 8,882 nm^2^ and TT_RTR_ is 1,937 nm^2^, Table 4), underscoring PTQ10’s ability in thick-film devices.

Yip’s team designed a novel fluorinated polythiophene derivative polymer donor, P4T2F-HD (Fig. 10d) [148]. It exhibits good planarity and has moderate miscibility with Y6-BO, promoting ideal nanoscale phase separation in the BHJ film (Fig. 10e), which is crucial for the charge-carriers transport. The P4T2F-HD:Y6-BO-based OPV achieved a high PCE of 13.65% with an active layer thickness of 110 nm. When the thickness increased to 300 nm, the PCE only decreased slightly to 12.48%, with a TT of 17,863 nm^2^ and TT_RTR_ of 3,500 nm^2^ (Table 4). The Gao’s group reported a small-molecule donor, ZW1, featuring dithiazole and thiophene in the π-bridge, with a planar structure (Fig. 10f) [149]. Its crystallinity enhances molecular packing and face-on orientation when added to the D18:Y6 binary system, thereby improving charge transport and balance. For the ternary devices, the *μ*_h_ is 5.27 × 10^–4^ cm^2^ V^−1^ s^−1^, the *μ*_e_ is 5.15 × 10^–4^ cm^2^ V^−1^ s^−1^, and the *μ*_h_/*μ*_e_ is 1.02, showing more balanced carrier transport compared to the binary system (Tables 4 and 5). Consequently, the ternary devices achieved PCEs of 18.50% at a thickness of 120 nm and 16.67% at 300 nm, with a TT of 15,510 nm^2^ and TT_RTR_ of 3,146 nm^2^ (Table 4).

#### Vertical Phase Distribution

Electrons and holes transport along the pure phases of the acceptor and donor, respectively. Therefore, a vertical phase distribution with the bottom enriched in donor and the top in acceptor is significantly advantageous in tradition OPVs. By forming a gradient phase distribution in the vertical direction, direct charge-carrier transport paths can be created, ensuring more efficient transport of electrons and holes to their respective electrodes, thereby enhancing the overall performance of thick-film devices [97, 98, 150–153].

**Fig. 11:**
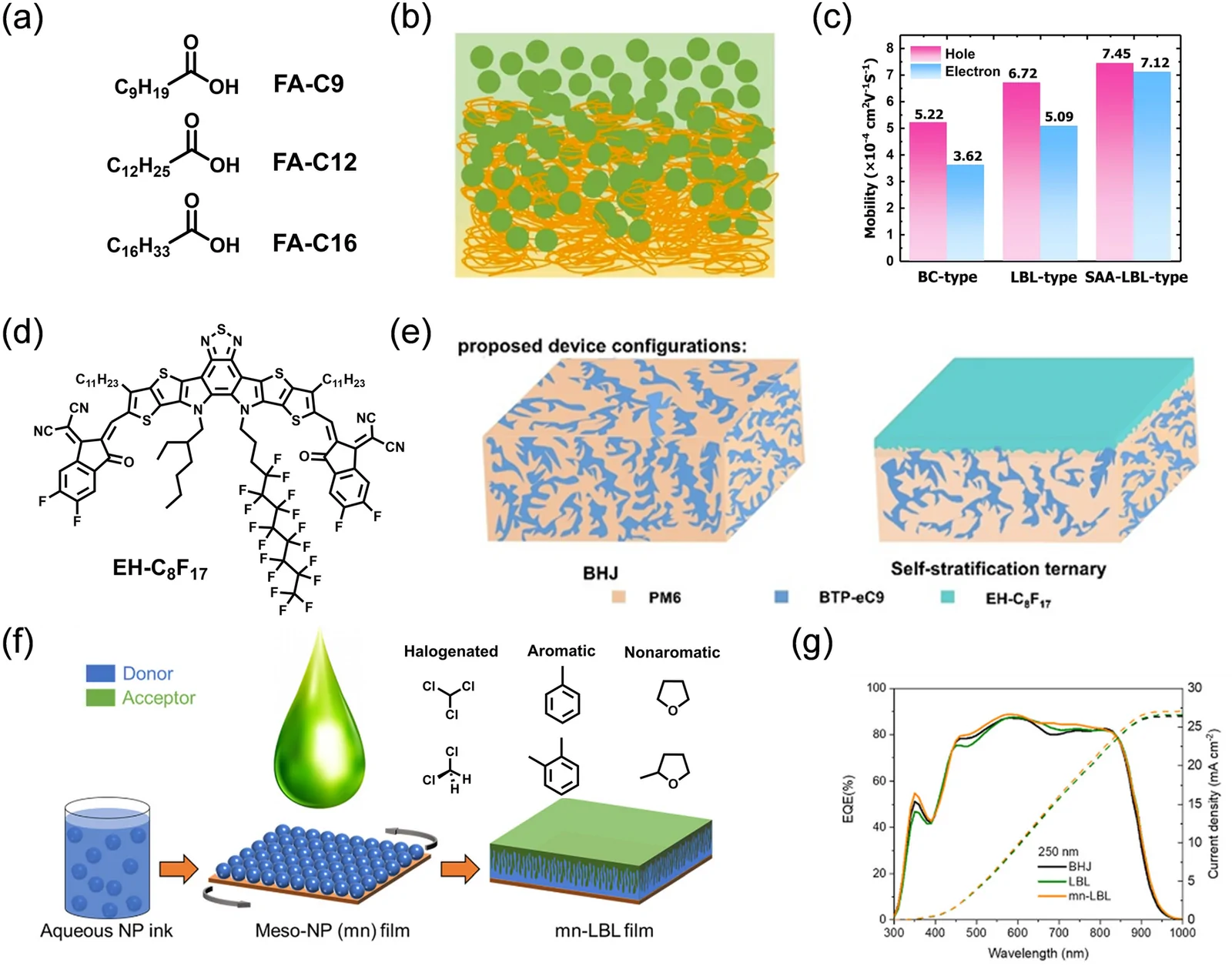
**a** Chemical structure of three FAs (FA-C9, FA-C12, FA-C16). **b** Diagram of solid additive-assisted LBL-type morphology. **c** *μ*_h_ and *μ*_e_ of three devices based on PM6:Y6 tested from SCLC. **b–c** are reprinted with permission from [154], copyright 2023 Springer Nature. **d** Chemical structures of EH-C_8_F_17_. **e** Schematic illustration of BHJ and self-stratification ternary devices. **e** is reprinted with permission from [155], copyright 2023 Wiley–VCH. **f** Schematic diagram of the mesostructured-nanoparticle (mn)-based LBL polymer:NFA film processed by water and solvents for processing the acceptor layer. **g** EQE spectra for various devices of 250 nm thickness. **f–g** are reprinted with permission from [156], copyright 2024 The Royal Society of Chemistry

Chen et al. reported a solid additive-assisted LBL strategy, addressing the issue of uncontrollable acceptor diffusion in traditional LBL techniques [154]. The addition of a solid additive (fatty acid, FA, as shown in Fig. 11a) to the donor PM6 not only formed more precise phase separation but also promoted controllable diffusion of the acceptor during the LBL process, creating an ideal vertical phase separation that enhances charge-carrier transport (*μ*_e_ = 7.12 × 10^–4^ cm^2^ V^−1^ s^−1^, *μ*_h_ = 7.45 × 10^–4^ cm^2^ V^−1^ s^−1^) and collection efficiency (Fig. 11b, c, Tables 4 and 5). As a result, the device (PM6:L8-BO) with a thickness of 250 nm achieved high PCE of 16.44%, with a TT of 5,813 nm^2^ and TT_RTR_ of 1,843 nm^2^ (Table 4). Huang’s team reported a self-stratification strategy, compared to traditional LBL processes, which greatly simplifies the preparation of complex ternary active layers and ensures an excellent vertical distribution, promoting charge-carrier transport and balance: *μ*_e_ is 7.91 × 10^–4^ cm^2^ V^−1^ s^−1^, *μ*_h_ is 9.79 × 10^–4^ cm^2^ V^−1^ s^−1^, and *μ*_h_/*μ*_e_ is 1.24 (Table 4 and Table 5), and improving the FF and *J*_SC_ of thick-film devices [155]. They introduced a novel fluorinated alkyl acceptor, EH-C_8_F_17_ (Fig. 11d), into PM6:BTP-eC9, achieving self-stratification in the vertical phase (Fig. 11e), successfully fabricating thickness-insensitive high-performance OPVs. Ultimately, a ternary device with an active layer thickness of 350 nm achieved a PCE of 16.89%, with a TT of 21,929 nm^2^ and TT_RTR_ of 5,536 nm^2^ (Table 4). Latterly, Li's group developed a LBL processing technique using aqueous nanoparticle (NP) inks containing donor materials [156]. This method constructed a highly packed mesoporous NP layer, which was further optimized by thermal treatment to ensure full penetration of the acceptor material and form an ideal vertical phase separation. Compared to traditional techniques, this strategy not only ensures thorough interdiffusion between the donor and acceptor but also uses thermal treatment to induce NP coalescence, limiting excessive deposition of the acceptor solution at the bottom, providing a more precise method for controlling vertical distribution (Fig. 11f, g). Due to efficient and balanced charge-carrier transport (*μ*_h_/*μ*_e_ = 1.36), as listed in Tables 4 and 5, PM6:BTP-eC9 OPV fabricated using the water-based NP-LBL technique achieved efficiencies exceeding 19.01%, with even thicker devices (18.2% at 250 nm and 17.2% at 400 nm) surpassing previously reported high-thickness OPVs, with a TT of 17,821 nm^2^ and TT_RTR_ is 4,248 nm^2^ (Table 4), facilitating the large-scale production of flexible, large-area devices.

#### Intermediate State Engineering

Intermediate state refers to transition phase formed during the film-formation process that can be induced by additives or specific post-treatment. Acting as templates or scaffolds, intermediate states guide the arrangement of molecules during the film formation process, resulting in a more ordered and crystalline structure [157, 158]. This enhances *π*-*π* stacking interactions between conjugated molecules, which is crucial for efficient charge transport. In well-ordered systems, the overlap of molecular orbitals is maximized, creating continuous pathways for charge carriers with minimal scattering or recombination losses [159]. This is especially important in thick films, where the longer charge travel distances to the electrodes make any disruption in transport pathways lead to significant efficiency losses. Moreover, controlling molecular packing through intermediate states allows for precise tuning of the film morphology, which can be optimized to meet the specific requirements of different device architectures and processing conditions [160, 161]. For example, in roll-to-roll printing or large-area fabrication, maintaining consistent, high-quality morphology across the entire film is essential for achieving uniform device performance.

**Fig. 12:**
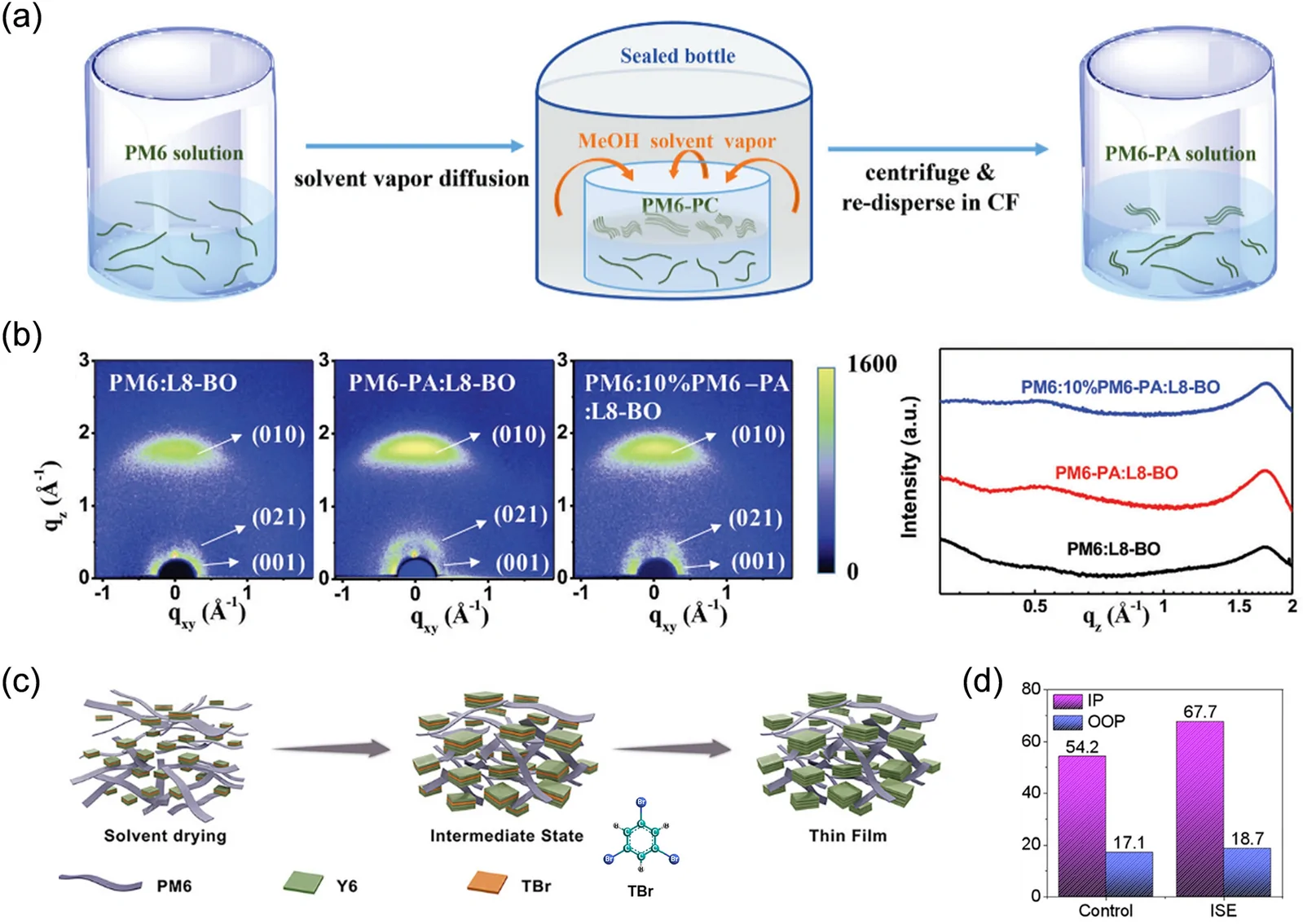
**a** Schematic illustration of the preparation of PM6-PC and PM6-PA solution. **b** 2D GIWAXS patterns and 1D GIWAXS profiles along out-of-plane of PM6:L8-BO, PM6-PA:L8-BO, and PM6:PM6-PA:L8-BO films. **a–b** are reprinted with permission from[162], copyright 2023 Wiley–VCH. **c** A schematic diagram of the ISE strategy. **d** The IP and OOP intensity of the control and ISE blend from the GIWAXS profiles. **c–d** are reprinted with permission from [163], copyright 2024 The Royal Society of Chemistry

In 2023, Wang’s team introduced a simple “polycrystalline-induced aggregation” strategy by preparing a PM6 pre-polymer (PM6-PA, as shown in Fig. 12a) in advance and incorporating it into the blended film [162]. This approach successfully produced high-efficiency devices with a film thickness exceeding 300 nm. By leveraging the polycrystalline nature of PM6, they significantly enhanced the molecular aggregation of the donor and acceptor materials L8-BO (Fig. 12b). This improved aggregation behavior influenced the structural order during film casting, promoting stronger molecular crystallization in the blended solution, which in turn enhanced charge-carrier transport (*μ*_e_ = 6.1 × 10^–4^ cm^2^ V^−1^ s^−1^, *μ*_h_ = 5.4 × 10^–4^ cm^2^ V^−1^ s^−1^, *μ*_h_/*μ*_e_ = 0.89) and collection in thick-film OPVs (Table 4 and Table 5). As a result, the device with a 300-nm-thick active layer achieved a PCE of 17.2%, with a TT of 9,523 nm^2^ and TT_RTR_ of 2,757 nm^2^ (Table 4). In the same year, Zhu’s group proposed an intermediate state engineering (ISE) strategy to fabricate efficient and thickness-insensitive OPV devices [163]. By introducing a solid additive, 1,3,5-tribromobenzene (TBr), they formed a TBr:Y6 intermediate phase (Fig. 12c), which promoted molecular self-aggregation and controlled phase separation. This optimized blend morphology facilitated stronger charge transport: *μ*_e_ is 1.34 × 10^–4^ cm^2^ V^−1^ s^−1^ and *μ*_h_ is 1.52 × 10^–4^ cm^2^ V^−1^ s^−1^ (Fig. 12d, Tables 4 and 5), improved charge collection, and reduced trap-assisted recombination. Devices based on the ISE strategy and PM6:L8-BO system demonstrated excellent thickness tolerance, with a 300-nm active layer achieving a champion PCE of 17.8%, with a TT of 15,384 nm^2^ and TT_RTR_ of 4,408 nm^2^ (Table 4).

TT and TT_RTR_ are useful for evaluating a system’s thickness tolerance. However, due to differences in material composition, systems with similar TT values may exhibit different TT_RTR_ values. For example, the PM6:Y6:BTP-M system has a higher TT value (9,464 nm^2^) than the PM6:L8-BO system (5,813 nm^2^), but its TTRTR value (1,919 nm^2^) is only slightly higher than the latter's (1,843 nm^2^). Conversely, while the TT value of PM6:Y6:BTP-M is slightly lower than that of PM6:PM6-PA:L8-BO (9,523 nm^2^), its TT_RTR_ value (1,919 nm^2^) is significantly lower than the latter’s (2,757 nm^2^). This indicates that material and process differences can lead to variations in TT_RTR_ even when TT values are similar. Therefore, the RTR concept is valuable for comparing systems with different efficiency benchmarks. Most thick-film devices have TT values on the order of 10^4^. The thick-film devices discussed in this paper are mainly based on non-fullerene acceptor systems. Fullerene systems, with their higher charge mobility, tend to have higher TT values, as reported in the literature [84]. TT values exceeding 100,000 nm^2^ are considered ideal. Such values enable devices to maintain high efficiency across a broad thickness range (e.g., from 100 to 300 nm) with a PCE drop of less than 20%. Moreover, devices with higher TT and TT_RTR_ values are more practical for large-scale production due to their lower sensitivity to thickness variations.

### Charge-Carrier Recombination and Extraction: Minimizing Losses

**Fig. 13:**
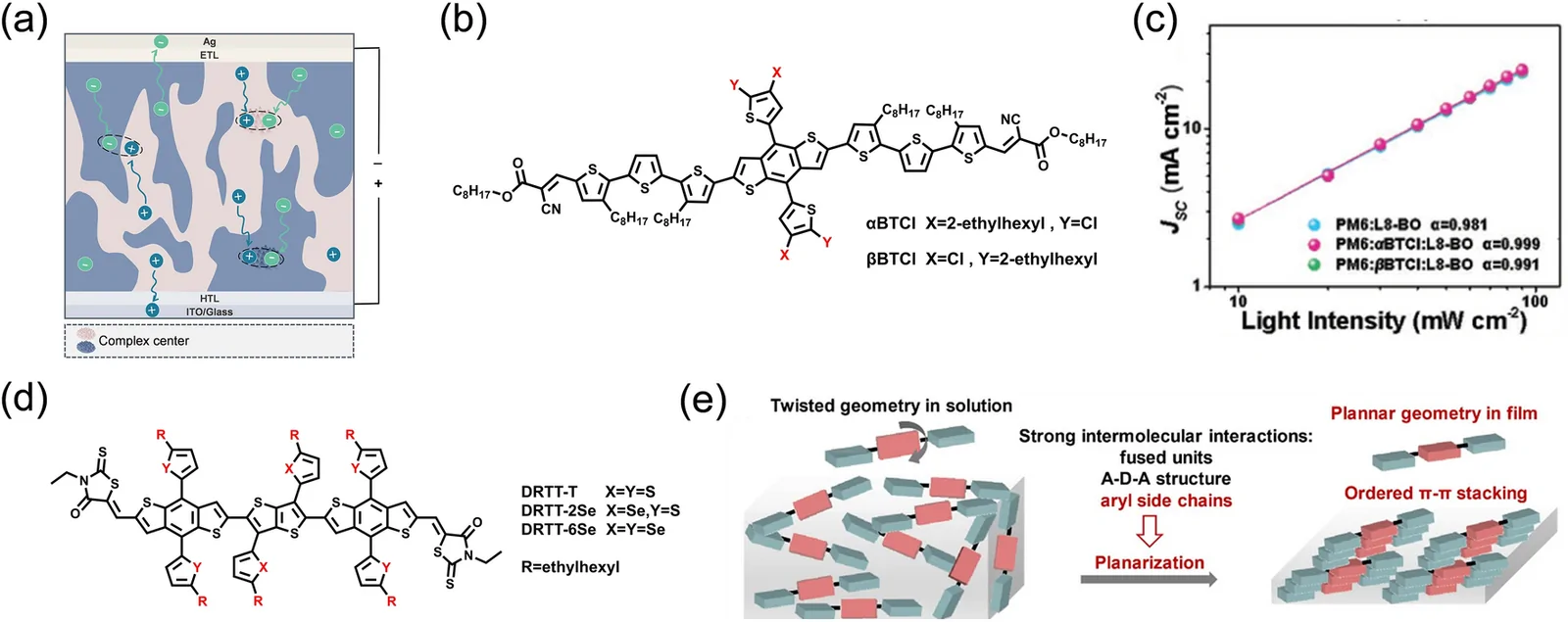
**a** Schematic illustration of charge-carrier recombination and collection. **b** Chemical structures of SM donors. **c** The light intensity dependence of *J*_SC_ of binary and optimized ternary devices.** c** is reprinted with permission from [174], copyright 2023 Wiley–VCH. **d** Chemical structures of DRTT-T, DRTT-2Se, DRTT-6Se. **e** Design strategy of the small molecule donors in the work. **e** is reprinted with permission from [175], copyright 2023 The Royal Society of Chemistry

In organic semiconductors, charge-carrier recombination mechanisms mainly include bimolecular recombination and trap-assisted recombination, as shown in Fig. 13a. Bimolecular recombination refers to the direct combination of two charge carriers (electrons and holes). This type of recombination typically occurs when the concentration of charge carrier is high, especially during charge-carrier transport when there is a space-charge accumulation region in the active layer [164–166]. In OPVs, the bimolecular recombination rate is discussed based on the Langevin model and can be expressed by the following formula:

8 $$\gamma_{{{\mathrm{bi}}}} = \frac{{q(\mu_{e} + \mu_{h} )}}{{\varepsilon_{r} \varepsilon_{0} \gamma }}$$

Herein, *q* represents the elementary charge, *μ*_e_ and *μ*_h_ are the mobilities of electrons and holes, respectively, *ε*_r_ is the relative dielectric constant of the material, *ε*_0_ is the dielectric constant of free space, and 1/*γ* is a pre-factor (*γ* ≫ 1) [167, 168]. This mechanism involves the trapping of charge carriers (electrons or holes) in trap states, followed by recombination with oppositely charged carriers in OPVs [169].

Increasing the charge-carrier extraction efficiency and reducing the density of defect states can both lower the charge-carrier recombination rate [170, 171]. The charge-carrier extraction length is defined as the ratio of the charge-carrier drift length (*L*_dr_ = $\mu \tau F$) to the active layer thickness (*d*), where (τ) is the carrier lifetime, μ is the charge-carrier mobility, and F = (*V*_bi_-*V*)/*d* is the electric field. Consequently, the charge-carrier collection efficiency decreases with increasing active layer thickness. By increasing the carrier mobility and carrier lifetime, the charge-carrier collection efficiency can be improved, thereby enhancing the photocurrent in thick-film devices.

The charge recombination dynamics of OPV is usually studied by measuring *J*-*V* curves under various light intensities (*P*_light_). The relationship between *V*_OC_ and *P*_light_ can be used to describe the trap-assisted recombination (*nk*T/*q*, where *q* is the elementary charge, *k* is the Boltzmann constant, T is the absolute temperature, and the *n* value represents the fitted slope; a higher *n* value indicates more severe trap-assisted recombination). The bimolecular recombination could be represented via the relation of *J*_SC_ ∝ *P*_light_^α^ (where *α* is the exponential factor, the closer *α* is to 1, the less bimolecular recombination there is) [172].

#### Liquid Crystal Donors

Side-chain design plays a crucial role in the development of OPV materials [173]. By modifying the side chains, it is possible to regulate the solubility, crystallinity, and intermolecular interactions of molecules, thereby affecting the morphology of the active layer and photovoltaic performance.

Alkyl aromatic rings are commonly used side chains for modulating molecular planarity and crystallinity. Molecules with chlorinated thiophene side chains typically exhibit higher molecular planarity and crystallinity, as well as more ordered liquid crystalline properties, which facilitate the formation of more uniform active layer morphologies. Wang and colleagues designed and synthesized two novel small molecule donors with *α*-chlorothiophene (*α*BTCl) and *β*-chlorothiophene (βBTCl) side chains, as shown in Fig. 13b [174]. These molecules, when added as a third component to the PM6:L8-BO binary blend, improved the active layer morphology and reduced charge-carrier recombination (Fig. 13c), successfully fabricating devices with a thickness exceeding 300 nm. The ternary film based on *α*BTCl achieved a record PCE of 17.46% at an active layer thickness of 330 nm, with a TT of 14,400 nm^2^ and TT_RTR_ of 4,171 nm^2^ (Table 6). Compared to alkyl thiophene side chains, alkyl selenophene side chains possess stronger intermolecular interactions. Moreover, these small molecules can effectively suppress charge-carrier recombination, thereby significantly enhancing the performances of thick-film devices. The Li’s team carefully regulated the molecular side chains to design two small molecule donors with twisted conjugated skeletons, DRTT-2Se and DRTT-6Se (Fig. 13d) [175]. These molecules exhibited strong intermolecular interactions and formed more ordered π-π stacking structures under appropriate annealing conditions without increasing phase separation size. This design helps reduce non-radiative recombination losses and energy disorder, thereby suppressing charge-carrier recombination (Fig. 13e) and achieving more efficient thick-film OPVs. Ultimately, binary devices based on DRTT-2Se:N3 (TT is 13,584 nm^2^) and DRTT-6Se:N3 achieved optimal PCEs of 13.2% and 13.81%, respectively, at an active layer thickness of 300 nm, with a TT of 17,704 nm^2^ and TT_RTR_ of 3696 nm^2^ (Table 6).

**Table 6:** Photovoltaic performances of OPVs with various film thickness and low charge-carrier recombination (discussed in this review)

Active layer	Thickness (nm)	*V*_OC_ (V)	*J*_SC_ (mA·cm^−2^)	FF (%)	PCE (%)	TT (nm^2^)	TT_RTR_ (nm^2^)	Refs.
PM6:αBTCl:L8-BO	90	0.904	26.73	78.52	18.96	14,400	4171	[174]
	330	0.873	27.28	73.24	17.46			
DRTT-2Se:N3	120	0.850	25.18	68.80	14.79	13,584	2791	[175]
	300	0.850	23.33	66.90	13.20			
DRTT-6Se:N3	120	0.850	25.29	69.90	15.03	17,704	3696	
	300	0.840	25.53	64.30	13.81			
PMQ-Si605:PM6:BTP-H2	110	0.907	27.18	77.69	19.15	7829	2095	[177]
	150	0.904	26.50	76.77	18.40			
	200	0.908	26.69	74.36	18.02			
PM6:BTP-eC9	100	0.860	28.00	80.50	19.40	10,000	2910	[180]
	300	0.850	28.40	72.30	17.40			
PM6:L8-BO	100	25.93	0.893	78.61	18.20	12,861	2926	[181]
	300	26.93	0.866	73.30	17.09			

#### Polymer Donor with Different Molecular Weight

The molecular weight (Mn) of polymer donor has a significant impact on the performance of thick-film OPVs. Polymers with higher Mn are beneficial for improving device PCEs, as well as enhancing the tolerance of the active layer thickness and thermal stability. Campoy-Quiles and coworker evaluated the relationship between the photovoltaic performance, active layer thickness tolerance, and thermal stability of OPV devices using donor polymers with different Mn [176]. Compared with low-Mn PTQ10 (such as 2.4 kDa), when the active layer thickness is up to 350 nm, the PCE of high-Mn PTQ10 (such as 52.9 kDa) can be increased by more than three times to 10.1%. The absorption peak of high-Mn PTQ10 is sharper, the conjugation length and molecular order are improved, and the light absorption capacity is enhanced. Meanwhile, the hole mobility of high-Mn PTQ10 significantly increased (from (1.76 ± 0.55) × 10^4^ cm^2^ V^−1^s^−1^ at 2.4 kDa to (2.45 ± 0.43) × 10^–3^ cm^2^ V^−1^ s^−1^ at 54.4 kDa), forming longer chain segments and better interchain links and promoting charge transfer. Its crystallinity decreases and shows a face-on orientation, which is conducive to the charge transfer within the plane. In addition, the glass transition temperature (T_g_) of high-Mn PTQ10 is higher (195.3 °C for 52.9 kDa and 181.6 °C for 2.4 kDa), enhancing thermal stability, reducing phase separation and morphological changes at high temperatures, and thereby improving the performance and stability of thick-film devices. These factors work together to enhance the efficiency and stability of thick-film devices based on high-Mn PTQ10.

Later, Chen et al. modified PTQ11 using siloxane-terminated side chains and successfully synthesized a new siloxane-containing polymer, PMQ-Si605. Compared to PTQ11 with a Mn of 28.3 kg mol^−1^, the randomly copolymerized PMQ-Si605 with minor siloxane decoration achieved a higher Mn of 51.1 kg mol^−1^ [177]. This modification not only increased the Mn but also enhanced the aggregation ability and charge transport, while reducing the number of trap states. As a result, the active layer based on PMQ-Si605:PM6:BTP-H2 exhibited a PCE of 19.15%. Even when the active layer thickness was increased to 150 and 200 nm, the devices still achieved high PCEs of 18.40% and 18.02%, respectively, with a TT of 7,829 nm^2^ and TT_RTR_ of 2,095 nm^2^ (Table 6).

#### Regulating Intermolecular Interactions

**Fig. 14:**
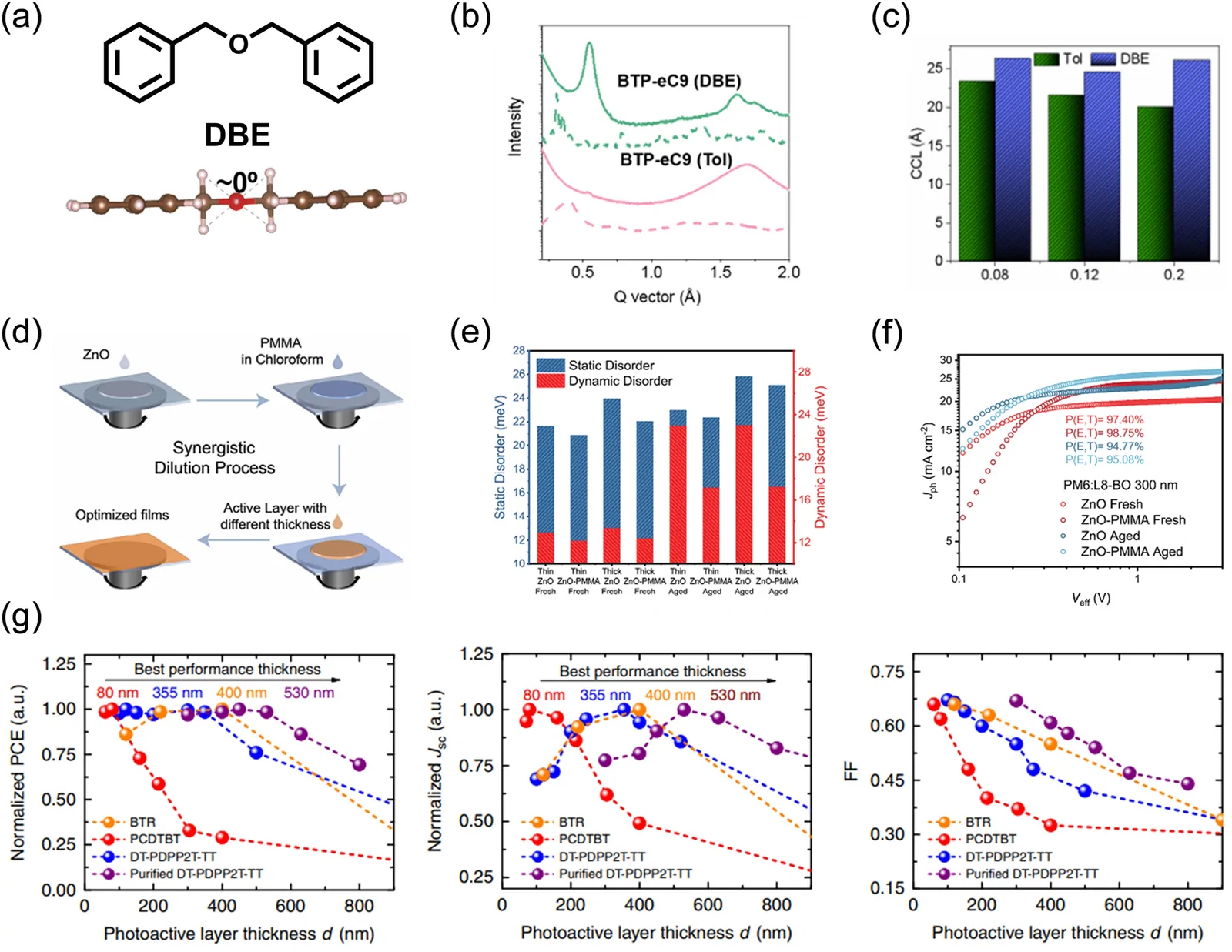
**a** Chemical structure of the additive. **b** In-plane and out-of-plane profiles of corresponding 2D GIWAXS patterns. **c** CCL calculated from the OOP (010) peak based on thick PM6:BTP-eC9 film without and with DBE additive. **a-c** are reprinted with permission from [180], copyright 2024 The Royal Society of Chemistry. **d** Schematic diagram of the processing process. **e** Comparison of static/dynamic disorder in different devices. **f** The evolution of *J*_ph_ versus *V*_eff_ for the fresh and aged 300-nm devices. **d-f** are reprinted with permission from [181], copyright 2024 Wiley–VCH. **g** Thickness-dependent device performance. PCDTBT:PC_71_BM, original DT-PDPP2T-TT:PC_71_BM, BTR:PC_71_BM and purified DT-PDPP2T-TT:PC_71_BM are compared with thickness-dependent device performance. **g** is reprinted with permission from [182], copyright 2019 Springer Nature

The strength of intermolecular interactions directly affects the kinetics of charge-carrier recombination. The enhancement of intermolecular interactions effectively suppresses the high energetic disorder and reduces the density of defect states within OPVs, ultimately leading to significantly improved photovoltaic performance and thickness tolerance [178, 179]. Song’s team proposed a method to introduce a non-halogenated dibenzyl ether (DBE, Fig. 14a) solvent additive into the PM6:BTP-eC9 system, inducing bifunctional hydrogen bonds and π-π intermolecular interactions with the acceptor (Fig. 14b), thereby obtaining a highly ordered polycrystalline structure [180]. This restructured morphology greatly reduced trap state density and energetic disorder (Fig. 14c), and thanks to improved charge-carrier recombination, the champion efficiency reached 17.4% when the active layer thickness was increased to 300 nm, with a TT of 10,000 nm^2^ and TT_RTR_ of 2,910 nm^2^ (Table 6).

A dilution strategy to suppress traps in both transport and active layers, by reducing molecular vibrations and optimizing intermolecular interactions to minimize static and dynamic disorder, also helps to inhibit charge-carrier recombination and enhance the efficiency of thick-film devices. The Hao’s group introduced poly (methyl methacrylate) (PMMA)-modified ZnO as an interfacial layer (Fig. 14d), effectively alleviating oxygen defects in ZnO and also regulating the self-assembly process of the active layer, achieving an orderly distribution of donors and acceptors, thereby reducing charge-carrier recombination traps (Fig. 14e, f) [181]. Consequently, the PM6:L8-BO device with a thickness of 300 nm showed a significant enhancement in photovoltaic performance after synergistic dilution, achieving a PCE of over 17%, and excellent thermal stability, with a TT of 12,861 nm^2^ and TT_RTR_ of 2,926 nm^2^ (Table 6).

#### Controlling Tail State Density

In thick OPVs, the presence of tail states and the depth dependence of photogenerated charges can work together to form space-charge-carrier layers, affecting charge-carrier extraction efficiency.

In 2019, Durrant and colleagues investigated the relationship between the photovoltaic performance of different absorber layer materials and the thickness of the absorber layer [182]. They found that as the active layer thickness increased, the PCE of most systems exhibited a severe thickness dependence (Fig. 14g). Upon analysis, researchers pointed out that the density and energy distribution of tail states are key factors limiting the collection of photo-current in thick OPVs. Therefore, in the process of fabricating large-scale devices with active layers exceeding 300 nm, it is essential to prioritize minimizing the tail state density in the active layer, rather than simply focusing on achieving more suitable mobility-lifetime (*μ*_τ_) product values. This work clearly explains the common decline in PCE of devices with thick active layers and provides a feasible approach to achieving high-performance thick-film devices.

## Scaling Up: Thick-Film Systems for Large-Area Applications

Thick-film OPV systems offer several advantages for large-area applications, including insensitivity to thickness variations, optimized exciton diffusion and dissociation, improved charge-carrier transport, better morphological control, and the ability to maintain high photovoltaic performance during large-area manufacturing. These advantages make thick-film OPVs a promising candidate for commercialization and high-performance development [183–188].

### Enhancing Exciton Diffusion and Dissociation

**Fig. 15:**
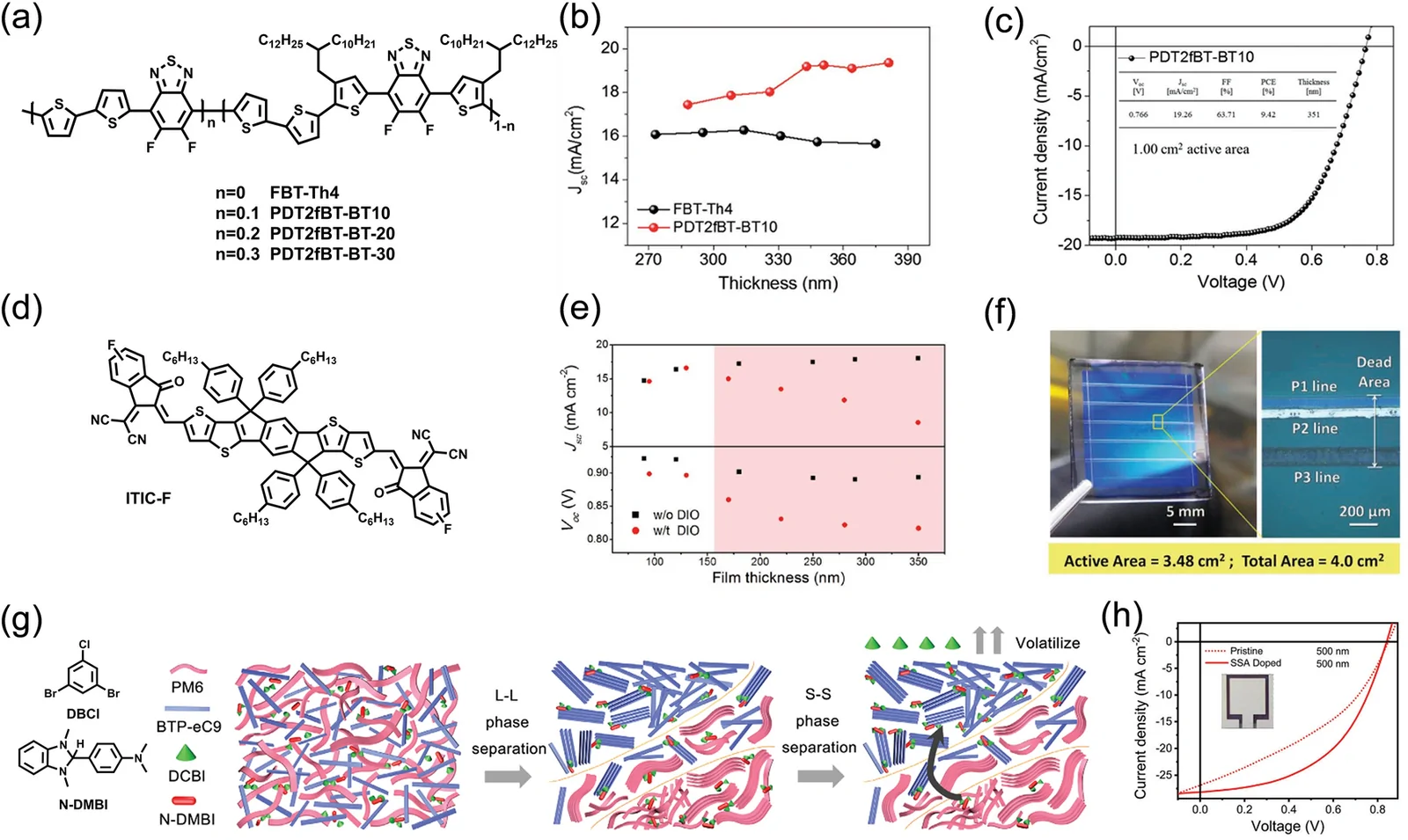
**a** Chemical structure of D1-A-D2-A type random ternary copolymer molecules. **b** Evolution of *J*_SC_ with the active layer thickness. **c** *J*-*V* curve of PDT2fBT-BT10 device (active area = 1 cm^2^, thickness of active layer = 351 nm). **b-c** are reprinted with permission from [189], copyright 2017 Wiley–VCH. **d** Chemical structure of ITIC-F. **e** *J*_SC_ and *V*_OC_ of OPVs based on PBTIBDTT:ITIC-F with varied film thicknesses processed without and with DIO. **f** Photograph of non-fullerene OPV modules and the optical micrograph of P1, P2, and P3 lines. **e–f** are reprinted with permission from [190], copyright 2018 Wiley–VCH. **g** Schemes of the dopant contained morphology evolution during the L-L and S–S phase separation stages. **h** *J*-*V* curves of 1-cm^2^ pristine and SSA-doped devices with 500 nm BHJ film thickness. The inset is the photograph of large-area device. **g–h** are reprinted with permission from [45], copyright 2023 Wiley–VCH

Optimizing material structures and the use of additives can significantly enhance the exciton *L*_D_ and dissociation efficiency. In 2017, Son et al. synthesized three D1-A-D2-A type random ternary copolymer molecules (Fig. 15a) [189]. Among these, PDT2fBT-BT10 demonstrated excellent processability and enabled the fabrication of uniform BHJ films with thicknesses ranging from 285 to 380 nm (Fig. 15b), even on large areas. PDT2fBT-BT10 exhibited high crystallinity with a predominantly face-on orientation, improving phase separation control. This led to enhanced exciton diffusion and charge-carrier transport. NA device based on PDT2fBT-BT10:PC_71_BM with an effective area of 1 cm^2^ and a thickness of 351 nm achieved a high PCE of 9.42% (Fig. 15c), with a TT of 28,202 nm^2^ and TT_RTR_ is 4,066 nm^2^. Next year, Yang’s team proposed a new combination of a wide-bandgap polymer, PBTIBDTT, and a narrow-bandgap small molecule acceptor, ITIC-F (Fig. 15d), successfully fabricating thickness-insensitive OPVs [190]. They found that the binary blend of PBTIBDTT:ITIC-F maintained similar morphologies and appropriate phase separation at different thicknesses (Fig. 15e), enabling efficient exciton dissociation and charge-carrier transport across various device thicknesses. Consequently, when the active layer thickness increased to 350 nm, the device maintained a PCE of over 9.0%, with a TT of 15,593 nm^2^ and TT_RTR_ of 2,145 nm^2^. Importantly, with an effective area of 3.48 cm^2^ (120 nm), the efficiency still reached a record-breaking 8.6% (Fig. 15f). In 2023, Yan’s group introduced a solid solvation-assisted (SSA) doping method, using 1,3-dibromo-5-chlorobenzene (DBCl) as a solid solute for N-DMBI, which was well miscible with the target components, successfully achieving component-selective electrical doping (Fig. 15g) [45]. Thanks to the longer exciton *L*_D_ and improved exciton dissociation efficiency, a binary device (PM6:BTP-e-C9) with a thickness of 500 nm achieved an impressive PCE of 15.55% (TT is 14,869 nm^2^ and TT_RTR_ is 3,390 nm^2^). Additionally, binary devices with effective areas of 1 cm^2^ and thicknesses of 100, 300, and 500 nm (Fig. 15h) achieved high PCEs of 16.10%, 14.24%, and 12.01%, respectively.

### Improving Charge-Carrier Transport

**Fig. 16:**
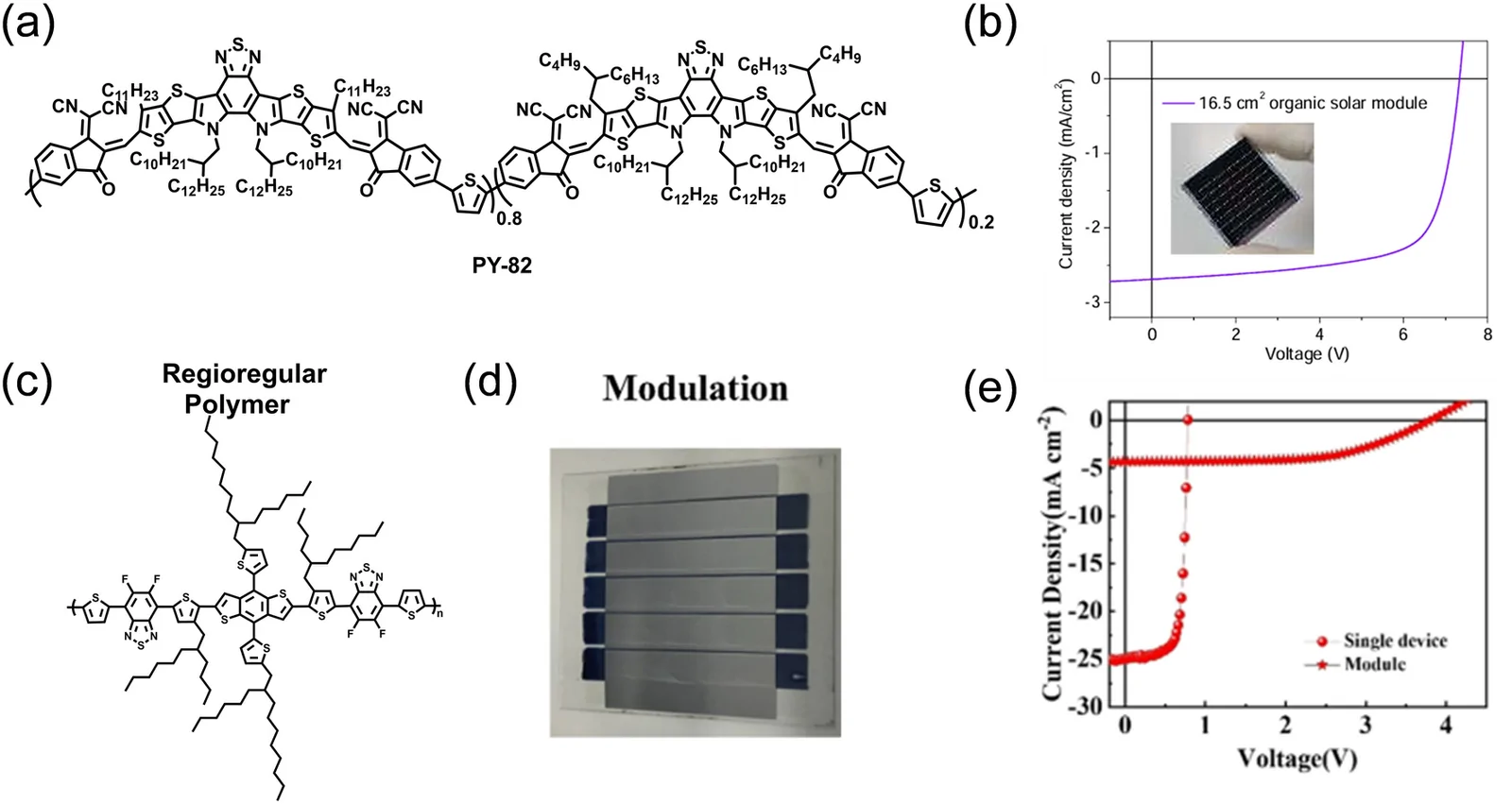
**a** Chemical structure of PY-82. **b** *J*-*V* curve of 16.5-cm^2^ module processed with o-xylene. **b** is reprinted with permission from [191], copyright 2022 Wiley–VCH. **c** Chemical structure of PDBD-2FBT. **d** Optical image of the module. **e** *J*-*V* characteristic for a single device and module. **d-e** are reprinted with permission from [192], copyright 2023 American Chemical Society

In 2022, Sun’s team developed a high-performance ternary all-polymer OPV by introducing PY-DT into PM6:PY-82 (Fig. 16a) [191]. The addition of PY-DT improved the film’s order and regularity, which enhanced charge-carrier transport and reduced energy disorder. Devices with a 300-nm-thick active layer achieved a PCE of 15.70%, with a TT of 8,772 nm^2^ and TT_RTR_ of 1,883 nm^2^, and large-area module (16.5 cm^2^) achieved a PCE of 13.84% (Fig. 16b). In 2023, Jin’s group synthesized a regioregular polymer, PDBD-2FBT (Fig. 16c), and paired it with Y6 as the acceptor, successfully fabricating binary devices with thick active layers and large areas [192]. The polymer exhibited strong regioregularity and high crystallinity after thermal annealing, which enhanced the device's tolerance to thicker films. This resulted in improved charge-carrier transport, with PCEs of 13.18% at 400 nm thickness and 9.55% in a 36 cm^2^ module (Fig. 16d, e), with a TT of 31,250 nm^2^ and TT_RTR_ of 5,892 nm^2^.

### Suppressing Recombination

**Fig. 17:**
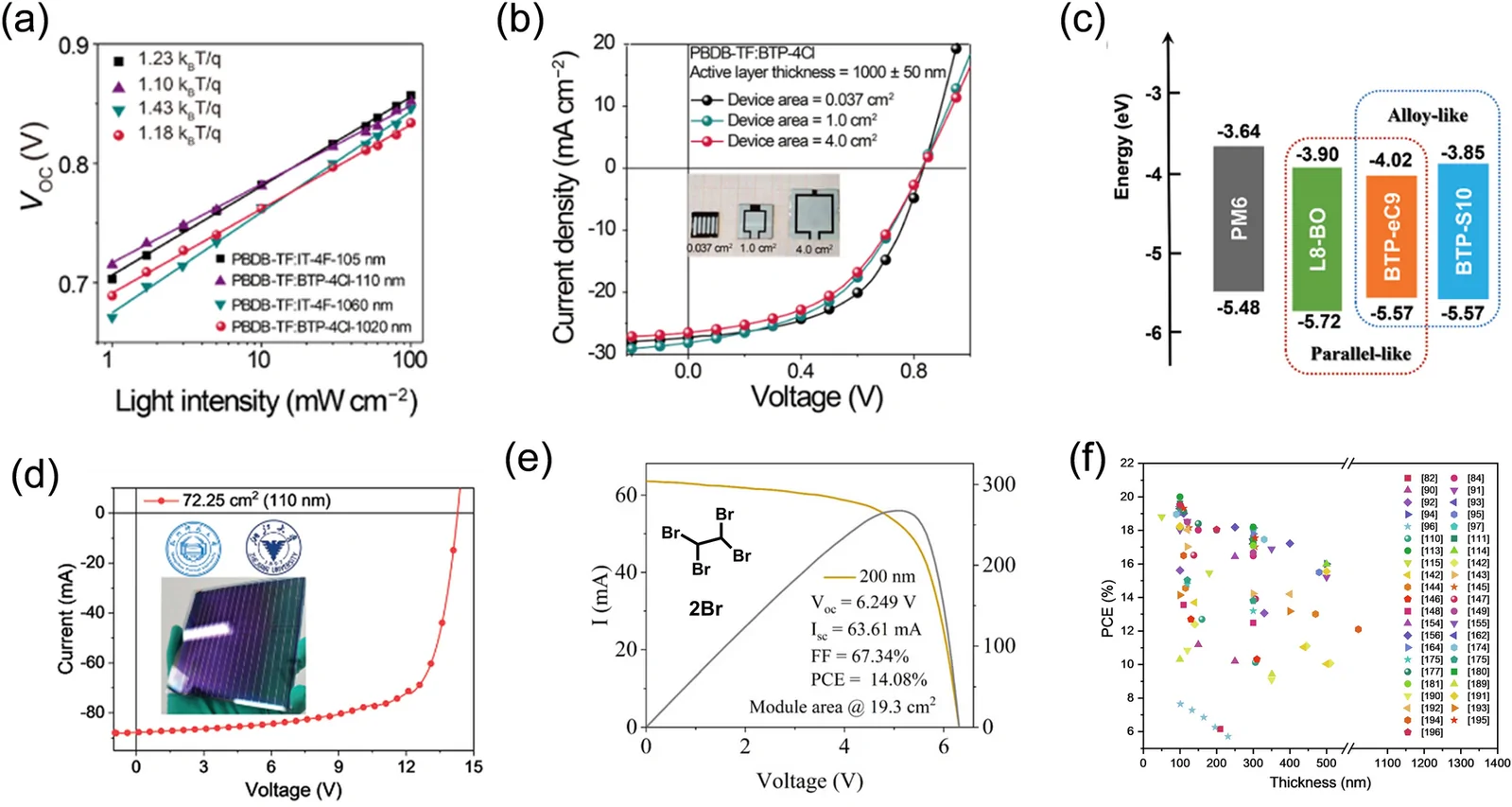
**a** *J*_SC_ vs light intensity of the PBDB-TF:IT-4F- and PBDB-TF:BTP-4Cl-based devices at different thicknesses. **b** *J*-*V* curves of the 1000-nm-thick BTP-4Cl-based devices with different areas; the inset shows the photographs of PBDB-TF:BTP-4Cl cells with different areas. **a-b** are reprinted with permission from [193], copyright 2020 American Chemical Society. **c** Energy diagram of PM6, BTP-eC9, L8-BO, and BTP-S10 determined by CV measurements. **d** *J*-*V* curves of the large-area devices with device areas of 72.25 cm^2^ (the insets are the photographs of the corresponding devices). **c–d** are reprinted with permission from [194], copyright 2022 Wiley–VCH. **e** *P*–*V* curves of the PM6:D18:L8-BO module with 200 nm thickness, inset is the chemical structure of 2Br.** e** is reprinted with permission from [195], copyright 2020 American Chemical Society. **f** Graph of the relationship between efficiency and active layer thickness (discussed in this review)

Reducing trap state density and suppressing recombination are crucial for the application of thick-film systems in large areas. In 2020, Hou et al. fabricated two types of thick-film devices using NFAs BTP-4Cl and IT-4F, along with the polymer donor PBDB-TF [193]. The devices based on the PBDB-TF:BTP-4Cl system exhibited lower trap state density (Fig. 17a). As the active layer thickness increased from 100 to 1,000 nm, the PCE decreased from 16.5 to 12.1%, with a TT of 22,750 nm^2^ and TT_RTR_ is 3,825 nm^2^. Additionally, for devices with an effective area of 1 cm^2^ and 4 cm^2^ and a thickness of 1,000 nm, high PCEs of 11.0% and 10.1% were achieved, respectively (Fig. 17b). Later, Zhan et al. reported a quaternary strategy to optimize the morphology of OPVs with multi-phase morphology (Fig. 17c), enhancing exciton separation and carrier mobility while reducing recombination and energy loss [194]. Ultimately, quaternary devices based on PM6:BTP-eC9:L8-BO:BTP-S10 achieved high efficiencies (up to 19.32% for 110-nm-thick layers and 17.55% for 305-nm-thick films, with a TT of 12,118 nm^2^ and TT_RTR_ is 3,329 nm^2^) and enables large-area devices (1.05 and 72.25 cm^2^, Fig. 17d) with efficiencies of 18.25% and 12.20%. The optimized intermixing-phase size supports thick-film and large-area applications, advancing OPVs practicality. In 2024, Lu’s team introduced a novel liquid additive, 2Br, which effectively regulated the aggregation behavior of NFAs by strengthening non-covalent interactions with the BTP core, thereby improving device performance [195]. Benefited from the suppressed trap-assisted recombination, the PM6:D18:L8-BO system with 2Br achieved a photovoltaic conversion efficiency of 18.05% at 200 nm, with a TT of 6,849 nm^2^ and TT_RTR_ is 2,673 nm^2^. When scaled up to a large-area module of 19.3 cm^2^ (Fig. 17e), it also achieved impressive PCEs of 15.66% (120 nm) and 14.08% (200 nm).

The magnitude of TT values serves as an indicator of the potential for OPV active layer materials in thick-film applications. However, high-efficiency thick-film OPV devices do not necessarily have the highest TT values (Fig. 17f), as material properties and fabrication processes also play roles. To achieve efficient and commercially viable OPV devices, it is crucial to balance these factors.

## Commercialization: Stability, Cost and Machine Learning

The commercial application of OPV devices holds significant environmental and social importance. Prior to this, we have thoroughly discussed the development status of high-efficiency thick-film OPVs and summarized a series of feasible device optimization methods. However, merely increasing the thickness of the active layer is not sufficient to achieve the widespread application of OPVs. At the same time, we should also consider addressing the stability of the devices and the cost of preparation and also the application of machine learning (ML) by artificial intelligence (AI) for predicting and analyzing the performance of material combinations.

### Stability

The relationship between active layer thickness and device stability is crucial, particularly in terms of the differing impacts on photothermal and mechanical stability. Studies reveal that variations in active layer thickness can significantly affect the stability profile of OPVs, with distinct mechanisms at play for each type of stability.

#### Photothermal Stability

In terms of photothermal stability, thick-film device seems to exhibit relatively poor performance. As highlighted in Min’s work, thick-film devices are prone to molecular aggregation and increased crystallinity under high-temperature conditions, leading to greater disorder in the active layer’s microstructure [196]. These microstructural changes can significantly impact the device’s optoelectronic properties. For instance, within the PM6:Y6 system, the device with a thickness of 150 nm shows its PCE dropping to only 35% of the initial value after being exposed to 150 °C for 48 h. In contrast, a device with a thickness of 32 nm can still maintain 92% of its initial performance under identical conditions. Further analysis indicates that the longer carrier transport pathways in thick films increase recombination probabilities. Coupled with heat-induced material degradation, these factors collectively result in the decline of photothermal stability.

Beyond this representative case, recent studies provide a more comprehensive understanding of the thickness-dependent degradation mechanisms in thick-film OPVs. Derya Baran et al. introduced a novel entropy-driven stabilization approach using a hexanary blend system (PM6 with five structurally similar *Y*-series NFAs) [188]. Devices featuring active layers up to 390 nm thick exhibited remarkable thermal stability, maintaining full performance after 552 h of thermal annealing at 130 °C in an inert atmosphere. This enhanced stability was attributed to the suppression of crystallization and phase separation due to increased configurational entropy, offering a promising pathway for scalable thick-film OPV modules. Yang’s team introduced DICO as a solvent additive to enhance the exciton LD in thick-film OPV, as mentioned in exciton behavior section [96]. The optimized morphology enabled stable operation in devices with 300-nm-thick active layers. Notably, DICO-treated devices retained over 90% of their initial power conversion efficiency (PCE) after 1,000 h at 85 °C, significantly outperforming the control devices (~ 70%). The improved operational stability was linked to reduced trap-assisted recombination, facilitated by the presence of DICO. Du’s research team investigated thick (100–400 nm thicknesses) PM6:BTP-eC9 devices fabricated via blade coating under ambient conditions [197]. Devices with a thickness of 120 nm retained 74% of their initial PCE after 200 h of light-thermal aging at 60 °C, whereas those with a 330 nm thickness dropped to 44%. The degradation was primarily attributed to the formation of trap states, which were closely associated with structural disorder and radical formation in PM6. Furthermore, Li et al. advanced thick-film stability by manipulating the crystallization sequence within the D18-Cl:N3 blend through the incorporation of a molecular regulator (AT-β2O) [184]. This strategy delayed N3 crystallization relative to D18-Cl, facilitating the formation of a vertically graded BHJ structure. Devices with 400-nm active layers maintained 86% of their initial PCE after 1,200 h of operation, compared to less than 60% for binary control devices without the regulator.

Recently, Hao’s team significantly enhanced the thermal stability of thick-film OPVs by promoting molecular order and reducing excited-state molecular distortion through interfacial dipole polarization and D-A dilution [198]. Their 300-nm-thick active layer devices retained about 80% of their initial PCE after 600 h of continuous thermal annealing at 100 °C. This strategy proved effective across multiple polymer donor:NFA systems, indicating its broad applicability in boosting stability for various OPV configurations.

Collectively, these results underscore the multifaceted mechanisms underlying thickness-dependent photothermal instability: from heat-accelerated radical formation and carrier recombination, to crystallization mismatch and vertical phase inhomogeneity. Simultaneously, strategies such as crystallization sequence regulation, phase structure engineering, and entropic stabilization provide viable pathways to suppress these degradation pathways, enabling thick-film OPVs with high efficiency and long-term stability.

#### Mechanical Stability

Regarding mechanical stability, thick-film devices demonstrate relatively good performance. For example, thick-film devices based on PNDI-2T (around 610 nm in thickness) can preserve their uniform morphology and photovoltaic performance after 100 bending cycles [199]. Additionally, by introducing functional additives and a third component, the mechanical stability of thick-film devices can be significantly enhanced. In the case of the PM6:PBB1-F:Y6-BO-4Cl and PM6:PBB1-F:BTP-eC9-based ternary flexible thick-film OPVs, the addition of PBB1-F and PAE allows the devices to retain over 90% of their performance after 1000 bending cycles with a diameter of 10 mm [6]. This improvement in mechanical stability is mainly attributed to enhanced intermolecular interactions and the role of functional additives. Specifically, the introduction of PBB1-F optimizes molecular stacking and strengthens intermolecular interactions, while PAE acts as a locking cage-like structure, effectively boosting the mechanical strength of the active layer.

In conclusion, the photothermal stability of thick-film devices is primarily limited by microstructural changes and the deterioration of carrier transport properties, whereas their mechanical stability benefits from enhanced intermolecular interactions. Future research should focus on developing strategies to address the thickness sensitivity in both stability aspects to further advance the commercialization of thick-film OPVs.

### Cost

In the competitive landscape of today’s market, cost is a pivotal factor influencing the commercial viability of any product. OPV devices are no exception. The cost structure of OPV devices encompasses active layer materials, buffer layer materials (including electron and hole transport layer materials), metal electrode materials, and transparent conductive glass substrates. Currently, the combined cost of these materials is relatively high, which somewhat diminishes the competitiveness of OPV devices in industrial applications [200–202].

However, thick-film OPV devices offer significant advantages in terms of material cost efficiency. Unlike traditional thin-film OPV devices, which may require multiple layers of materials for different components, thick-film OPV devices streamline material usage. For instance, fabricating three thin-film OPV devices (with 100 nm thickness) would typically necessitate three layers of buffer materials, three layers of metal electrode materials, and three layers of transparent conductive glass substrates. In contrast, a single thick-film OPV device (with 300 nm thickness) requires only one layer of each corresponding material. This reduction in material usage directly translates to a notable decrease in overall costs, partially alleviating the issue of high material expenses.

In addition to the structural cost benefits offered by thick-film OPV devices, the development and deployment of low-cost, synthetically simple active layer materials have further enhanced their commercial appeal. Among thick-film OPV materials, PTQ10 is the promising low-cost polymer donors [146]. Its simple chemical structure enables a two-step synthesis from inexpensive, commercially available precursors, offering high synthetic efficiency. You et al. developed an optimized synthetic route that successfully reduced the production cost of PTQ10 from $214.18 to $30.29 g^−1^, an 86% decrease, thus demonstrating the material’s great potential for large-scale, low-cost manufacturing [203]. Beyond PTQ10, an increasing number of studies have focused on low-cost, structurally simple, and scalable polymer donors with excellent photovoltaic performance. For example, Sun et al. introduce two low-cost polymer donors, PTQ14 and PTQ15, with a trifluoromethyl (CF_3_) substituent. They cost $35/g, $0.36/W_p_, 1/6 the cost of other high-performance polymer donors [204]. PTQ15-based ternary OPV achieve a PCE of 19.96%. Additionally, the traditional (E)-2-[2-(thiophen-2-yl)vinyl]thiophene (TVT) unit maintains molecular planarity through a simple vinylene bond structure, and its synthesis and purification are straightforward, which greatly cuts the cost of organic semiconductor materials. By introducing fluorine atoms and ester groups to the TVT unit, Wei’s research team synthesized two new D-A copolymers, PBTVT-1 and PBTVT-2 [205]. Notably, PBTVT-2, with excellent solubility and a favorable molecular stacking mode, exhibits outstanding photovoltaic performance and high thickness tolerance. The introduction of this novel electron-withdrawing TVT unit is expected to significantly advance the development of low-cost, high-efficiency, thick-film OPVs. Moreover, a recent systematic economic analysis estimates that the manufacturing cost of fully OPV modules could range from $48.8 to $138.9 m^−2^ [206], translating to a levelized cost of $1.00–2.83/W_p_ (assuming 5% module efficiency). Importantly, incorporating low-cost donor materials such as PTQ10-series or TVT-based polymer donor could help bring these estimates toward the lower end of the range. Therefore, the synergy between material simplification and structural integration in thick-film OPVs represents a promising strategy not only for improving efficiency and stability but also for substantially reducing the overall device cost.

While thick-film OPV devices still face challenges in enhancing efficiency, their cost-control advantages are becoming increasingly evident. With ongoing technological advancements and innovations, we anticipate that thick-film OPV devices will achieve a balance of high performance and low costs. This progress will enable them to stand out in the competitive market and contribute significantly to the renewable energy sector.

### Machine Learning

As research on thick-film OPV devices continues to advance, the efficient screening and design of high-performance active layer material combinations have become a key issue. In recent years, AI, as a powerful technology for mining relationships within big data, has brought significant convenience and development to the scientific research field.

Li et al. reported on AI predicting organic active layer materials. They developed a universal automated model capable of rapidly predicting the PCEs of OPV devices and validated its accuracy through experiments, with an error less than 2% [207]. The model leveraged a graph neural network (GNN) architecture to establish the natural correspondence between molecular structure and properties, addressing the challenge that traditional computational methods could not directly obtain accurate device efficiencies. In the following year, a research team from the Hong Kong Polytechnic University introduced a novel graph transformation framework called RingFormer, specifically designed to capture the atomic and ring structures within OPV molecules to achieve accurate prediction of OPV performance [208]. Evaluated on the Clean Energy Project Database (CEPDB), RingFormer achieved a 22.77% relative improvement over the nearest competitor. This study not only enhanced the efficiency of screening organic optoelectronic materials and reduced computational costs but also provided strong support for the design and optimization of optoelectronic devices.

However, research on AI predicting thick-film active layer materials has been relatively limited. Recently, the Campoy-Quiles’ group proposed a method for predicting and analyzing the performance of material combinations in thick-film OPV using high-throughput experimentation (HTE) and ML [209]. By analyzing 720 inverted devices and 20 different D-A combinations, they classified these materials into two major categories: thickness-sensitive and thickness-tolerant. They also revealed the key factors for achieving high FF in thick-film devices, including complementary absorption characteristics and balanced weight ratios. This approach offers new ideas and tools for designing and screening OPV materials suitable for thick-film applications.

It is evident that AI technology is playing an increasingly important role in global OPV research. It not only accelerates the discovery and performance optimization of new materials but also provides new perspectives and solutions for long-standing scientific challenges. Looking to the future, with the continuous development of AI technology, there is great potential to further drive the innovation and commercial application of thick-film OPV materials.

## Conclusions

In summary, advancements in material design, synthesis, and device engineering have optimized the dynamic behaviors of excitons and charge carriers, driving significant progress in thick-film OPVs. To achieve thickness-insensitive devices for large-scale production, careful optimization of device parameters is necessary to balance light harvesting, excitons diffusion and generation. Increasing film thickness is essential for scalability and enhancing light absorption, especially in semiconductors with low absorption coefficients. However, this also introduces challenges in effective exciton diffusion and dissociation. Strategies such as using low-bandgap materials, solvent additives, and controlling molecular packing can improve exciton dynamics and phase separation. Simulations of light-field distribution and electrical characteristics play a key role in fine-tuning these parameters for maximum efficiency, ensuring both scalability and high-performance thick-film OPVs. Additionally, enhancing and balancing charge-carrier mobilities are essential for thickness-insensitive OPVs. Materials that enable efficient charge transfer, characterized by high planarity, structural rigidity, small twist angles, and strong face-on aggregation, should be prioritized for future commercial applications. Improving phase purity, vertical phase separation, and charge-transfer pathways through morphology control are also effective strategies for enhancing charge mobility and device performance. Furthermore, reducing recombination rates, as seen in several systems compared to Langevin predictions, is crucial for efficient charge collection, benefiting both thick-film performance and furthering the understanding of charge-carrier dynamics in OPVs. Closing the efficiency gap between high-performance lab-scale devices and large-scale modules requires overcoming inherent losses from module structures, processing conditions, and material inhomogeneities. Investigation the stability loss mechanism, the cost benefits, and the application of ML method for thick-film OPVs. Achieving this will necessitate continued research to better understand and optimize the mechanisms involved in thick-film OPVs fabrication for commercial production.
