# Correction: Optimizing Exciton and Charge-Carrier Behavior in Thick-Film Organic Photovoltaics: A Comprehensive Review

**DOI:** 10.1007/s40820-025-01884-0

**Published:** 2025-08-06

**Authors:** Lu Wei, Yaxin Yang, Lingling Zhan, Shouchun Yin, Hongzheng Chen

**Affiliations:** 1 Key Laboratory of Organosilicon Chemistry and Materials Technology of Ministry of Education, Zhejiang Key Laboratory of Organosilicon Material Technology, College of Materials, Chemistry and Chemical Engineering, Hangzhou Normal University, 311121 Hangzhou, People’s Republic of China; 2 State Key Laboratory of Silicon and Advanced Semiconductor Materials, Department of Polymer Science and Engineering, Zhejiang University, 310027 Hangzhou, People’s Republic of China

**Correction to**: **Nano-Micro Letters (2026) 18:10**. 10.1007/s40820-025-01852-8

Following publication of the original article [1], the authors reported that the last author’s name was inadvertently misspelled. The published version showed “Hongzhen Chen”, whereas the correct spelling should be “Hongzheng Chen”.

The correct author name has been provided in this Correction, and the original article [1] has been corrected.
